# Co-stimulation with IL-1β and TNF-α induces an inflammatory reactive astrocyte phenotype with neurosupportive characteristics in a human pluripotent stem cell model system

**DOI:** 10.1038/s41598-019-53414-9

**Published:** 2019-11-15

**Authors:** Tanja Hyvärinen, Sanna Hagman, Mervi Ristola, Lassi Sukki, Katariina Veijula, Joose Kreutzer, Pasi Kallio, Susanna Narkilahti

**Affiliations:** 1 NeuroGroup, Faculty of Medicine and Health Technology, Tampere University, Tampere, Finland; 2 Micro and Nanosystems Research Group, Faculty of Medicine and Health Technology, Tampere University, Tampere, Finland

**Keywords:** Lab-on-a-chip, Neuroimmunology, Extracellular recording, Induced pluripotent stem cells

## Abstract

Astrocyte reactivation has been discovered to be an important contributor to several neurological diseases. *In vitro* models involving human astrocytes have the potential to reveal disease-specific mechanisms of these cells and to advance research on neuropathological conditions. Here, we induced a reactive phenotype in human induced pluripotent stem cell (hiPSC)-derived astrocytes and studied the inflammatory natures and effects of these cells on human neurons. Astrocytes responded to interleukin-1β (IL-1β) and tumor necrosis factor-α (TNF-α) treatment with a typical transition to polygonal morphology and a shift to an inflammatory phenotype characterized by altered gene and protein expression profiles. Astrocyte-secreted factors did not exert neurotoxic effects, whereas they transiently promoted the functional activity of neurons. Importantly, we engineered a novel microfluidic platform designed for investigating interactions between neuronal axons and reactive astrocytes that also enables the implementation of a controlled inflammatory environment. In this platform, selective stimulation of astrocytes resulted in an inflammatory niche that sustained axonal growth, further suggesting that treatment induces a reactive astrocyte phenotype with neurosupportive characteristics. Our findings show that hiPSC-derived astrocytes are suitable for modeling astrogliosis, and the developed *in vitro* platform provides promising novel tools for studying neuron-astrocyte crosstalk and human brain disease in a dish.

## Introduction

Astrocytes are the most abundant cell type in the central nervous system (CNS) and play important roles in supporting neuronal cell functions by releasing energy substrates and trophic factors, maintaining homeostatic functions and regulating neuronal synaptogenesis and synaptic transmission^1–4^. During CNS injury and disease, astrocytes transform into a reactive phenotype with changed morphology and altered gene expression and secretion profiles^4,5^. These astrocytes are regarded as immunocompetent cells that can respond to complex inflammatory events by secreting cytokines and chemokines, thereby controlling immune cell activation and migration to sites of damage^5,6^. Astrocytes are considered to play a dual role in CNS pathophysiology, as they can both support regeneration and exert detrimental effects on surrounding cells and brain parenchyma^6,7^. Interestingly, two distinct astrocyte phenotypes were recently described in rodent studies: a detrimental A1 type and a beneficial A2 type^8,9^. Of these, A2 astrocytes were associated with ischemia-induced CNS lesions, while A1 astrocytes dominated in inflammation-induced lesions reflecting those in diseases such as multiple sclerosis (MS). However, reactivation of astrocytes is a complex phenomenon that possibly results in a mixture of the two phenotypes or even several distinct yet unidentified activation states^10^.

The majority of the knowledge on reactive astrocytes has been gathered from rodent studies, whereas the inflammatory nature of human astrocytes has received less attention^9–11^. Nevertheless, growing evidence shows significant structural and functional differences between human and rodent astrocytes, underlining the relevance of further studies focusing on human astrocytes and their behaviors^12–14^. *In vitro* studies with human cells have typically utilized astrocytes from primary fetal or adult tissues or immortalized astrocytoma cell lines^14–17^; however, the availability of human primary astrocytes is limited, and immortalized cells are often criticized as model systems. Advancements in the field of stem cell biology have enabled the differentiation of astrocytes from human pluripotent stem cells (hPSCs)^18–22^, providing an unlimited cell source and an option to create disease-specific cell lines. Despite these advancements, only a few studies have described the reactivation of hPSC-derived astrocytes^18,22–25^, and even fewer studies have reported the interplay of inflammation or reactive astrocytes with human neuronal cells^24,26,27^.

The development of newly engineered *in vitro* platforms based on hPSC-derived cells is a promising approach for studying the mechanisms of CNS functions and disorders^28–30^. Microfluidic devices are potent research tools for studying the interactions of several cell types in controlled, compartmentalized culture environments^31,32^. For example, multiple cell compartments can be connected via microtunnels, allowing the growth of axons while restricting neuronal somas thus facilitating experimentation on cellular processes and cell-to-cell interactions^31^. Advantages in microfluidic technology have already been validated, for instance, in axonal transport and myelination studies^33–39^. However, less data are available on neuron-astrocyte interactions and neuroinflammatory activity^26,31^. Understanding the complex cellular interplay among several neural cell types can help to reveal underlying mechanisms in neurodegenerative diseases and create opportunities for drug discovery.

Here, we studied reactivation using human induced pluripotent stem cell (hiPSC) -derived astrocytes and observed their transformation into cells with reactive proinflammatory phenotypes with neurosupportive characteristics. Furthermore, we designed a novel microfluidic co-culture platform including neurons, astrocytes and an inflammatory environment that was validated for studying reactive astrocyte-neuron interactions. We are convinced that hiPSC-astrocyte model systems can facilitate investigation of specific human cell properties and ultimately model human CNS diseases in a dish.

## Results

### Characterization of hiPSC-derived astrocytes

**Figure 1:**
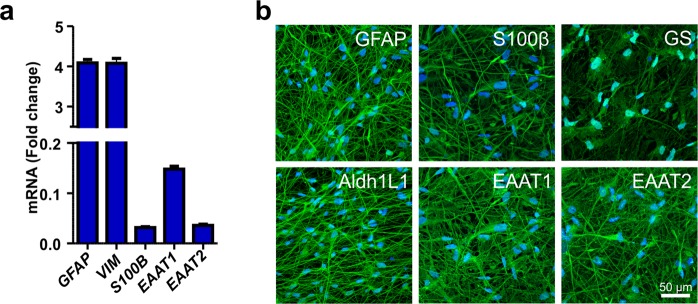
Characterization of hiPSC-derived astrocytes. (**a**) The hiPSC-derived astrocytes expressed several astrocyte-specific genes, including *GFAP, VIM, S100B, EAAT1* and *EAAT2*. The data are presented as the mean ± s.e.m. (n = 2 with three technical replicates; the data are representative of two experiments). (**b**) Immunocytochemical staining was used to confirm the expression of the major astrocyte markers GFAP, S100β, GS, Aldh1L1, EAAT1 and EAAT2.

HiPSC-derived astrocytes were first characterized in their quiescent resting state for the expression of astrocyte-specific markers at the gene and protein levels. Gene expression analysis revealed expression of transcripts typical for developing astrocytes, including S100 calcium-binding protein beta (*S100β*) and vimentin (*VIM*) (Fig. 1a). Additionally, mature astrocyte genes, such as glial fibrillary acidic protein (*GFAP*) and excitatory amino acid transporters 1 and 2 (*EAAT1* and *EAAT2*, respectively), were expressed (Fig. 1a). Major astrocyte-specific markers were also confirmed by immunocytochemical staining of GFAP, S100β, glutamine synthetase (GS), aldehyde dehydrogenase 1 family member (Aldh1L1), EAAT1 and EAAT2 (Fig. 1b). To confirm that the astrocytes were mostly negative for neuronal markers, the cells were stained for microtubule-associated protein 2 (MAP2) (Supplementary Fig. S1). Thus, the culture consisted of a heterogeneous astrocyte population with a cell type-specific expression signature.

### Treatment of hiPSC-derived astrocytes with IL-1β and TNF-α induces a reactive phenotype

**Figure 2:**
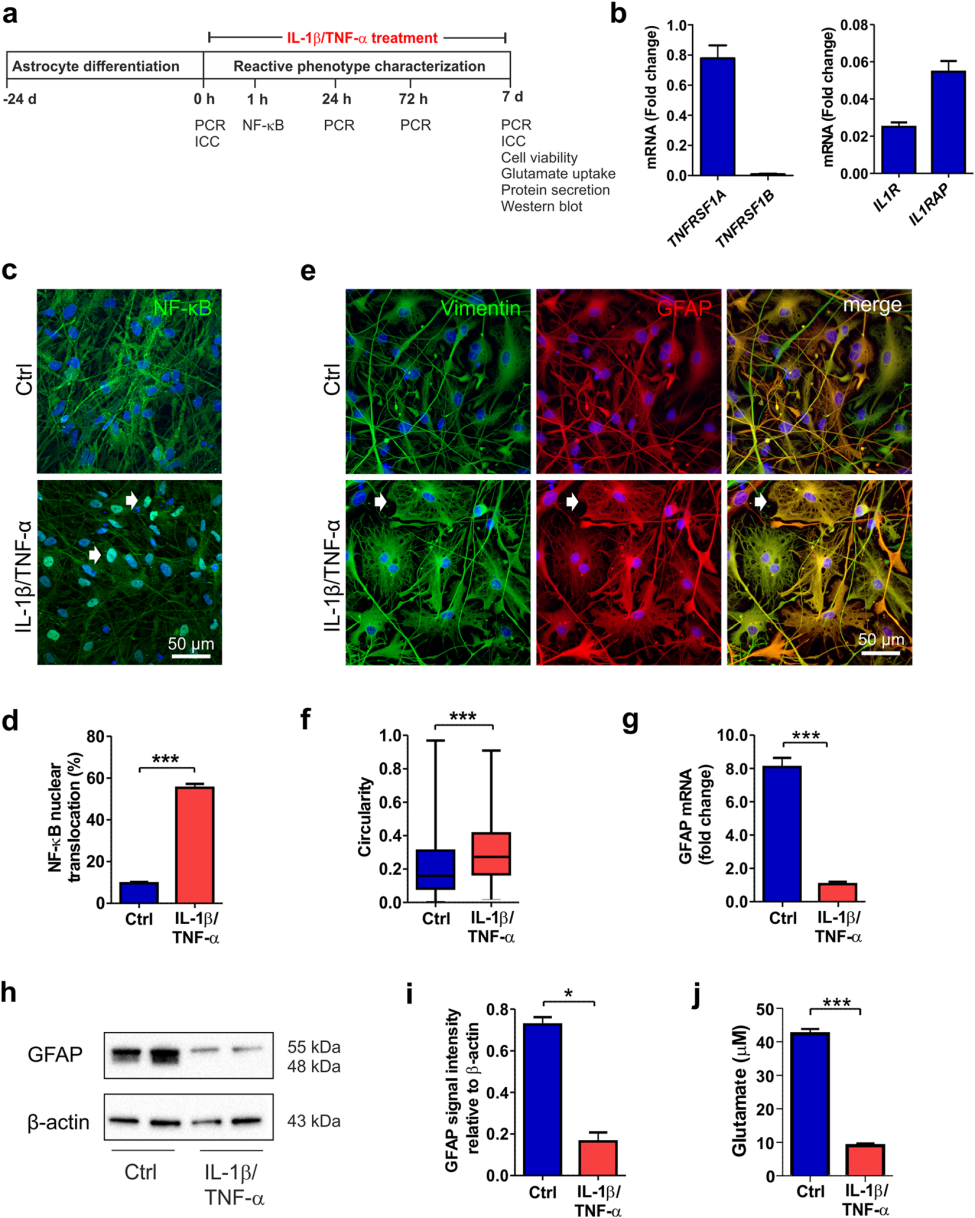
IL-1β and TNF-α treatment induces a reactive astrocyte phenotype. (**a**) Experimental setup for astrocyte stimulation with IL-1β and TNF-α and astrocyte characterization. (**b**) Gene expression analysis revealed the presence of TNFRSF1A, IL1R, and IL1RAP transcripts in the astrocytes (n = 2 with three technical replicates; the data are representative of two experiments). (**c**) In control astrocytes, NF-κB was ubiquitously expressed in the cytoplasm, whereas after 60 minutes cytokine stimulation, it was activated and translocated to the nucleus (white arrows). (**d**) NF-κB activation was quantified as the percentage of nuclei with translocation among the total nuclei (n = 6 cultures; the data were analyzed from two experiments). (**e**) Immunocytochemical staining of the intermediate filament proteins vimentin and GFAP showed morphological changes from filamentous to flattened shapes (white arrows) in response to cytokine stimulation. (**f**) Morphological change was quantified based on the vimentin staining of samples on day 7. The circularity of the cells in each group is presented as the median and interquartile range, with the whiskers showing the minimum and maximum values. Images were analyzed from six cultures derived from three experiments. (**g**) *GFAP* expression levels decreased following cytokine stimulation (n = 2 with three technical replicates; the data are representative of two experiments). (**h**) GFAP was analyzed at the protein level by western blot analysis, representative cropped images of the membranes for the GFAP and β-Actin are shown here and full length of membranes are presented in the Supplementary Fig. S4. (**i**) Quantification of GFAP and β-Actin showed a reduction after cytokine treatment. β-Actin was used as a loading control (n = 2; the data are representative of three experiments). (**j**) Cytokine treatment impaired the glutamate uptake capacity of astrocytes (n = 3; the data are representative of two experiments). The data are presented as the mean ± s.e.m. Statistical analysis was performed with independent-sample t-tests except in (**f**), in which statistical analysis was performed with the Mann-Whitney U test. Statistical significance: * p < 0.05, ** p < 0.01, and *** p < 0.001.

To induce a reactive astrocyte phenotype with inflammatory cytokines and to characterize astrocyte properties, an extended seven-day co-stimulation with IL-1β and TNF-α was performed (Fig. 2a). First, the presence of cytokine receptors for IL-1β and TNF-α in hiPSC-derived astrocytes was analyzed in the quiescent resting state. The expression of IL-1 receptor 1 (*IL1R*) and IL-1 receptor accessory protein (*IL1RAP*), as well as that of tumor necrosis factor receptor superfamily 1 A (*TNFRSF1A*), which is important for mediating the signals of these cytokines, was confirmed (Fig. 2b). However, *TNFRSF1B* was not detectable in the hiPSC-derived astrocytes (Fig. 2b).

Nuclear factor kappa B (NF-κB) is a key transcription factor that has been shown to be activated in reactive astrocytes during neuroinflammation^6^. Here, NF-κB was ubiquitously present in the cytoplasm of quiescent astrocytes but was only present in low amounts in the nuclei (Fig. 2c). However, soon after the initiation of cytokine treatment, NF-κB was activated and translocated to the nucleus (Fig. 2c, Supplementary Fig. S2). After 60 min of cytokine stimulation, a significant portion (over 50%) of the astrocytes showed activation of the NF-κB pathway (control [Ctrl] 9.7 ± 0.6 vs. IL-1β/TNF-α treated [Trt] 55.3 ± 1.9, p < 0.001, Fig. 2d).

Reactive astrocytes undergo changes in morphology typically characterized by hypertrophy of main cellular processes^40^. Over the 7-day cytokine treatment period, the astrocytes gradually exhibited a morphological change from a highly filamentous shape to a flattened polygonal appearance (Supplementary Fig. S3). This typical response was most notable upon the staining of the intermediate filament proteins vimentin and GFAP, both of which contribute to astrocyte morphology (Fig. 2e). The morphology of the control and treated astrocytes was quantified based on vimentin staining at the 7-day time point (Fig. 2f). The circularity index was calculated according to the area and perimeter metrics of each cell, with a value of 1 describing a perfectly circular object. Quantification confirmed that the astrocytes lost their filamentous shape and adopted a more circular morphology in response to cytokine activation (medians Ctrl 0.16 vs. Trt 0.27, p < 0.001, Fig. 2f).

Changes in intermediate filament protein expression have often been considered to be hallmarks of reactive astrocytes^10^. Although the cytokine-stimulated reactive astrocytes showed positive staining for GFAP (Fig. 2e), we observed an 8-fold decrease in mRNA levels after the 7-day treatment in the treated cells compared to the controls (Ctrl 8.81 ± 0.54 vs. Trt 1.05 ± 0.15, p < 0.001, Fig. 2g). The GFAP protein levels were also confirmed by western blot analysis, showing similar decreases as a result of IL-1β and TNF-α treatment (Ctrl 0.73 ± 0.04 vs. Trt 0.16 ± 0.06, p = 0.010, Fig. 2h–i, Supplementary Fig. S4). Additionally, the vimentin protein levels were studied and were found to remain more stable than GFAP protein levels after cytokine treatment (Supplementary Fig. S4).

Uptake of glutamate from the extracellular space is an important function of astrocytes that sustains healthy synaptic transmission of neurons^4^. Impairment in the uptake of glutamate from the culture medium was revealed in cytokine-stimulated astrocytes compared to control astrocytes (Ctrl 42.4 ± 1.4 vs. Trt 9.0 ± 0.66, p < 0.001, Fig. 2j). In summary, treatment with the cytokines IL-1β and TNF-α activated astrocytes, modified their intermediate filament cytoskeleton and transformed them morphologically and functionally into cells with a reactive phenotype.

### Cytokine treatment induces astrocyte proliferation and reduces viability

**Figure 3:**
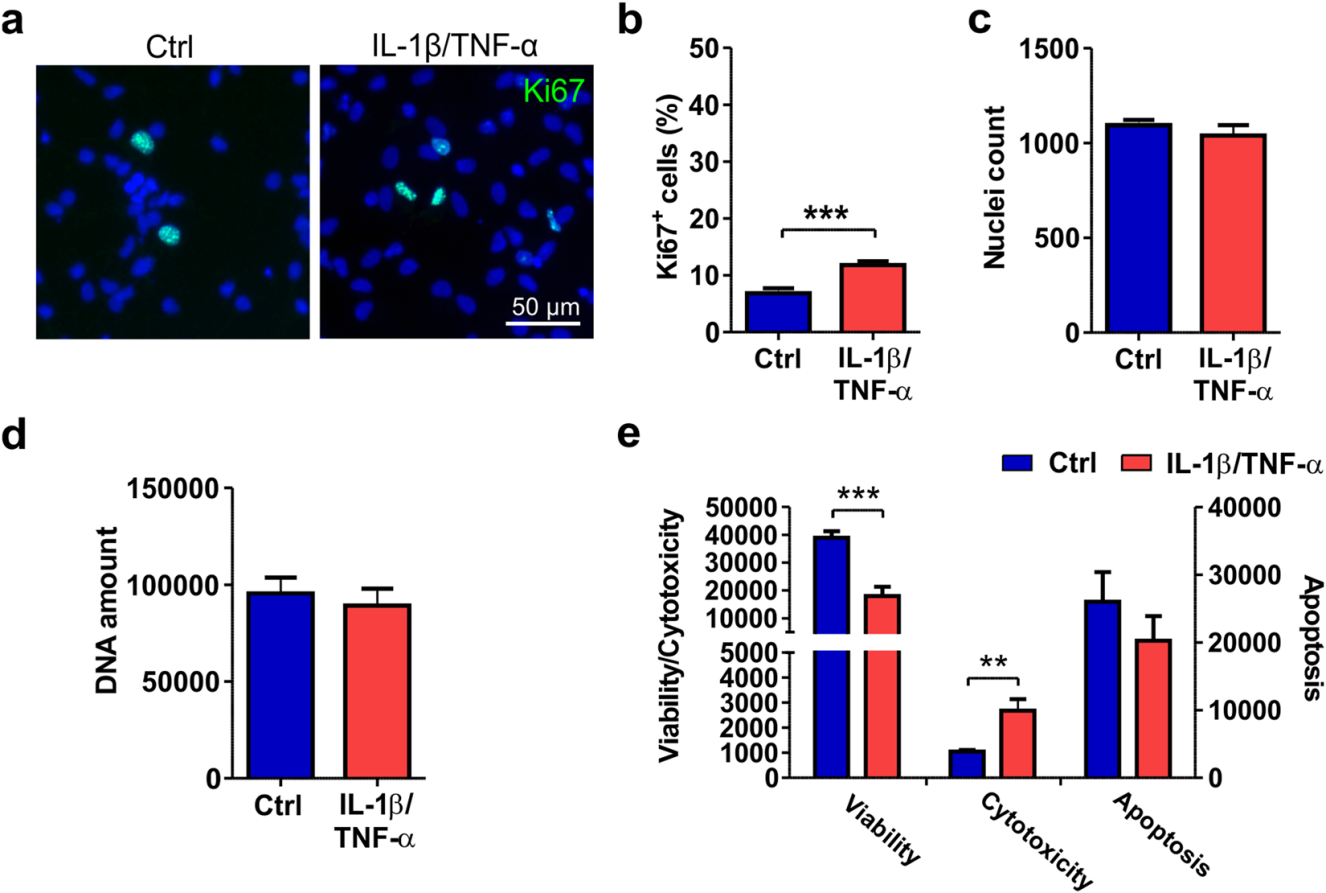
Viability and proliferation of cytokine-treated astrocytes. (**a**) Immunocytochemical staining of proliferating Ki67-positive cells in control and IL-1β/TNF-α**-**treated cultures. (**b**) The number of proliferating cells was increased after cytokine treatment. (**c**) The nuclei count quantified from DAPI staining remained similar between the control and treated groups. For Ki67 staining and nuclei counts, images were analyzed from 11 cultures derived from four experiments. (**d**) Additionally, the amount of DNA was similar between the groups (n = 6 with two technical replicates; the data are from two experiments). (**e**) The viability assay showed decreased viability and increased cytotoxicity but no significant difference in apoptosis following cytokine treatment (n = 3; the data are representative from two experiments). The quantification data are presented as the mean ± s.e.m. All statistical analysis was performed using independent-sample t-tests. The significant p-values are presented in the graphs. Statistical significance: * p < 0.05, ** p < 0.01, and *** p < 0.001.

Next, we studied the effects of cytokines on the viability and proliferation of astrocytes after a 7-day treatment regimen. Immunocytochemical staining with the proliferation marker Ki67 showed a slight (5%) but significant increase in proliferation in cytokine-treated cells compared to control cells (Ctrl 6.8 ± 0.9 vs. Trt 11.8 ± 0.7, p < 0.001, Fig. 3a–b). However, this increase did not significantly affect the total nuclei count (Ctrl 1094 ± 28 vs. Trt 1040 ± 55, Fig. 3c) or the total DNA amount (Ctrl 95476.7 ± 8299.5 vs. Trt 89263.8 ± 8779.2, Fig. 3d). Upon assessing the viability of astrocytes after cytokine treatment, a significant increase in cytotoxicity (Ctrl 1053.0 ± 55.8 vs. Trt 2677.3 ± 461.2, p = 0.017) and a decrease in viability (Ctrl 38938.2 ± 2347.3 vs. Trt 17999.2 ± 3352.4, p < 0.001) in the treated cells compared to the control cells were found (Fig. 3e). In the apoptosis metrics, no statistically significant differences were detected between the groups (Ctrl 26026.8 ± 4362.3 vs. Trt 20265.8 ± 3629.6, Fig. 3e). In conclusion, cytokine treatment of hiPSC-derived astrocytes caused minor proliferation, but the total cell number remained stable due to cytotoxicity.

### Reactivation induces a proinflammatory response in astrocytes

**Figure 4:**
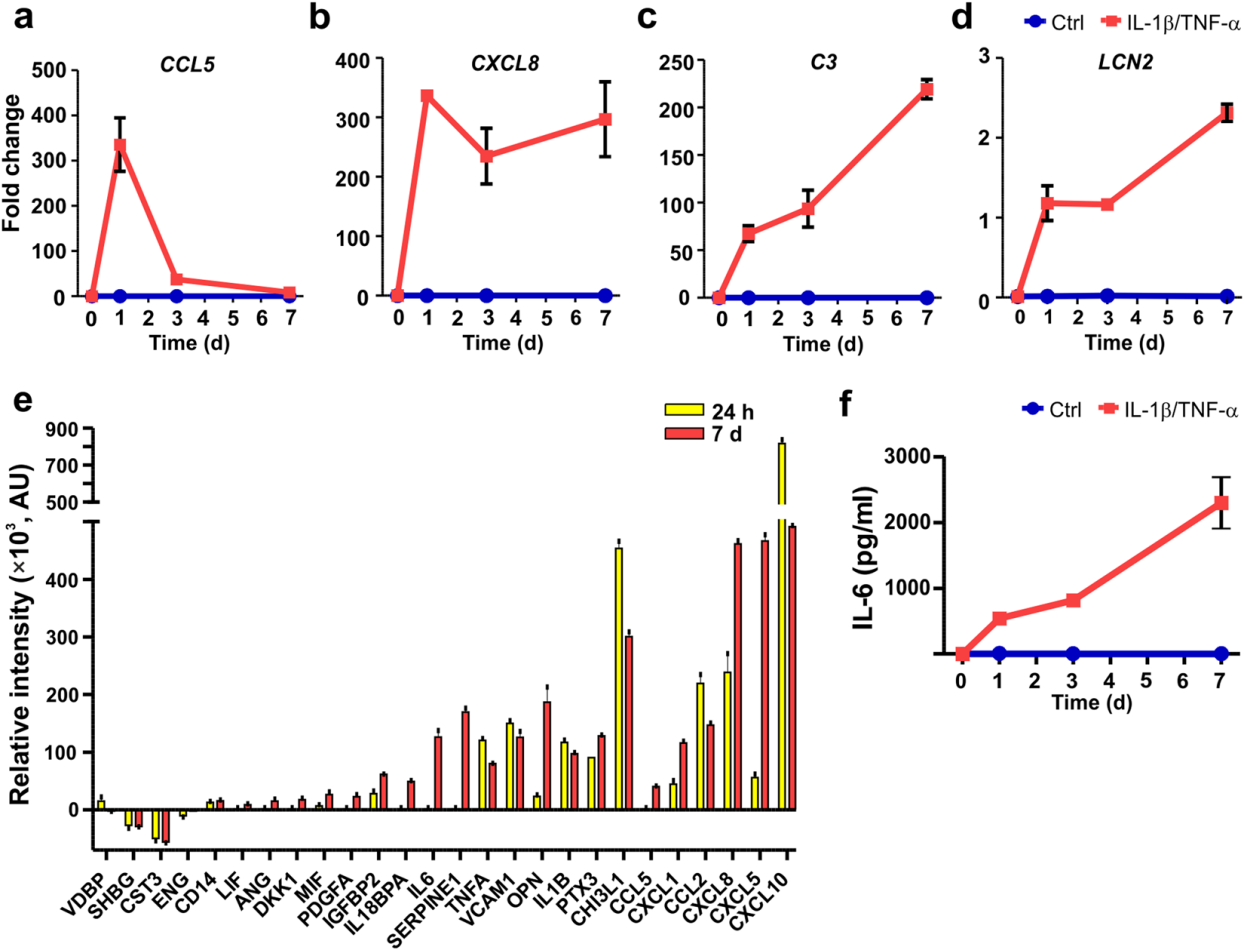
Cytokine treatment results in the inflammatory activation of astrocytes. (**a–d**) The gene expression levels of (**a**) *CCL5*, (**b**) *CXCL8*, (**c**) *C3*, and (**d**) *LCN2* were analyzed in cytokine-treated and control astrocytes at the 0-, 1-, 3- and 7-day time points. Robust increases in the expression of all studied genes were detected after cytokine treatment (n = 2 with three technical replicates; the data are representative of two experiments). (**e**) Secretion of inflammatory factors into the medium was analyzed at the 24 h and 7-day time points. The relative intensity values for the different analytes were calculated by subtracting the intensity values of the control samples from those of the cytokine-treated samples at each time point (n = 1 with two technical replicates; the data are representative of two experiments). (**f**) The levels of IL-6 were analyzed at 0, 1, 3 and 7 day in cytokine-treated and control astrocytes. IL-6 levels increased in a time-dependent manner over 7 days in the cytokine-treated samples, while under control conditions, the levels were undetectable (n = 2 with two technical replicates; the data are representative of two experiments). The quantification data are presented as the mean ± s.e.m.

To reveal whether the astrocytes were able to produce an immune response, the gene expression and secretion profiles of inflammatory markers were characterized. First, the effects of IL-1β and TNF-α treatment on the expression of the inflammation-related genes *CCL5*, *CXCL8*, complement component 3 (*C3*) and lipocalin-2 (*LCN2*) were studied (Fig. 4a–d). At the basal level, none of the transcripts were expressed, whereas treatment for 24 h led to over 300-fold increases in the expression of the chemokines *CCL5* and *CXCL8* (Fig. 4a–b). Recently, C3 has been linked to astrocyte reactivity^8,41^. Here, *C3* expression increased in a time-dependent manner after cytokine treatment, reaching over a 200-fold change compared to the control level at the 7-day time point (Fig. 4c). The gene expression of *LCN2* also considerably increased over 7 days, although the change was not as prominent as that for the other transcripts (Fig. 4d).

Next, the secretion of 105 inflammatory proteins (see full list in Supplementary Table 1) from cytokine-stimulated astrocytes was studied (Fig. 4e; Supplementary Fig. S5a). Treatment induced widespread protein secretion at the 24 h and 7-day time points (Fig. 4e and Supplementary Fig. S4a). The most highly secreted proteins were chemokines (CXCL10, CXCL5, CXCL8, CCL2, CXCL1, and CCL5), but other inflammatory factors, such as pentraxin 3 (PTX3), osteopontin (OPN), chitinase-3 like 1 (CHI3L1), Serpin E1 and adhesion molecule vascular cell adhesion protein-1 (VCAM1), were also secreted. Among the cytokines, IL-6, IL-1β, TNF-α and macrophage migration inhibitory factor (MIF) were detected on medium obtained from the cytokine-treated astrocytes.

IL-6 is a classic inflammatory mediator known to be secreted from astrocytes after reactivation^42^; therefore, the secretion of IL-6 was further investigated. Control astrocytes did not secrete IL-6, but cytokine-stimulated astrocytes gradually secreted IL-6, the levels of which reached 2000 pg/ml at the 7-day time point (Fig. 4f). In summary, astrocytes responded to cytokine treatment with changes in gene expression and broad secretion of proinflammatory factors.

### Astrocyte conditioned medium sustains neuronal viability and supports functional activity

**Figure 5:**
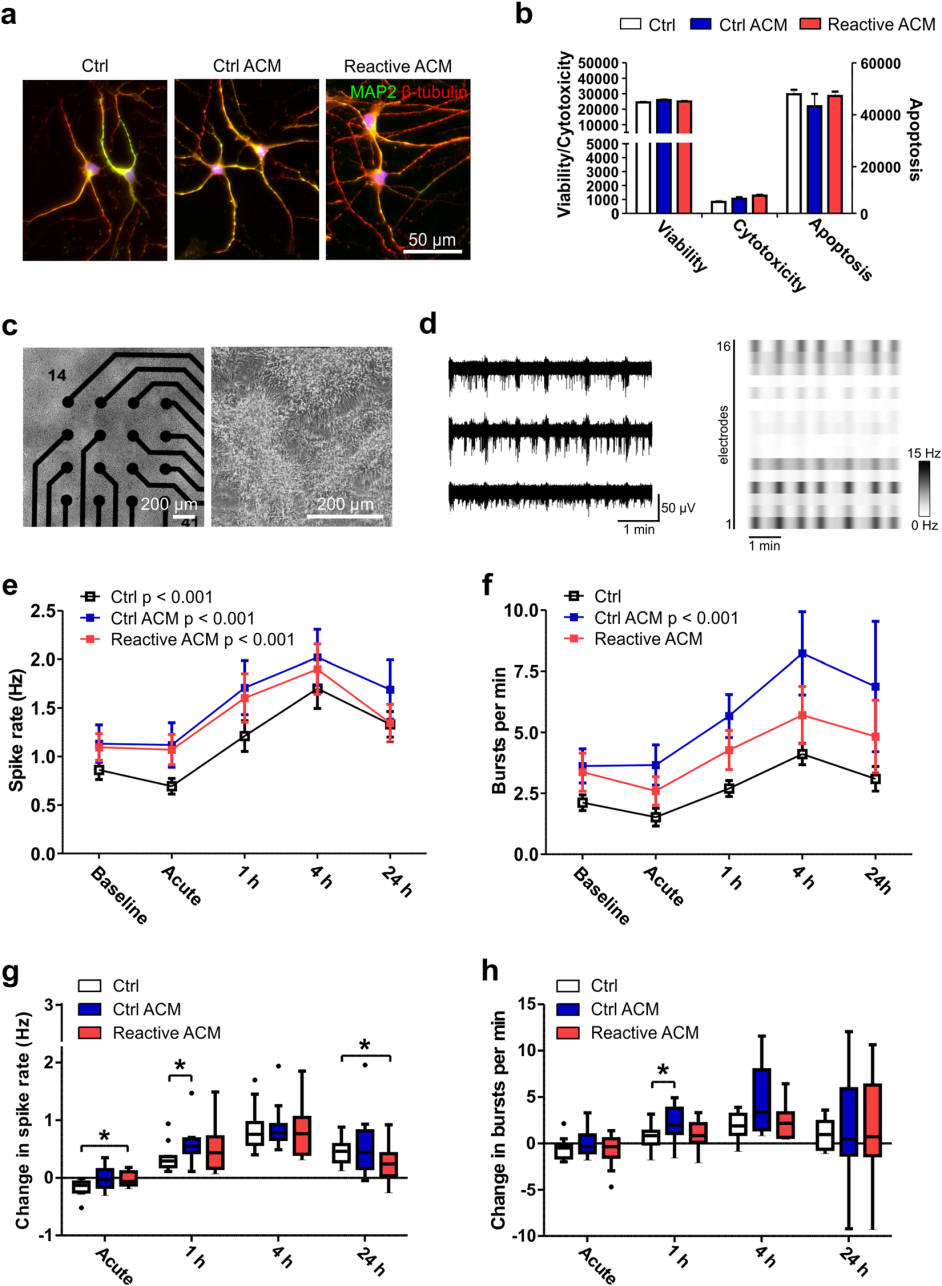
Viability and functionality of neuronal cells after exposure to reactive astrocyte conditioned medium. (**a**) Immunocytochemical staining of hPSC-derived neuronal cells exposed to control medium (Ctrl), control ACM (Ctrl ACM) and reactive ACM. Neuronal cells stained positive for MAP2 (green) and β-tubulin (red). DAPI nuclear staining is shown in blue. (**b**) The reactive astrocyte conditioned medium did not show an effect on the viability, cytotoxicity or apoptosis of neuronal cells after 48 h of exposure (the data are representative of two experiments; in each experiment n = 3–6 per group). Statistical analysis was performed using independent-sample t-tests. (**c**) The effect of ACM on neuronal functionality was studied with microelectrode array (MEA) measurements. The phase-contrast images show neuronal cells cultured on MEAs. (**d**) Typical spontaneous activity in neuronal networks recorded from three electrodes over 5 minutes. The raster blot shows the typical network-wide activity from all 16 electrodes of one well over 5 minutes. (**e**) Spike rate (Hz) development for active electrodes before (baseline) and after different medium exposures (acute, 1 h, 4 h and 24 h). (**f**) Number of bursts per minute before and after different medium exposures. For the MEA data, n = 12 networks derived from one differentiation. The data are presented as the mean ± s.e.m., and statistical analysis was performed using the Friedman test. (**g-h**) To highlight significant differences between treatment groups, the data from panels e-f were reused and are presented as the changes in spike and burst rates compared to the baseline measurements. The data are shown as Tukey boxplots. Statistical significances was determined with the Mann-Whitney U test; * p < 0.05, ** p < 0.01, and *** p < 0.001.

Astrocyte activation resulted in widespread secretion of inflammatory molecules; therefore, we next studied whether astrocyte conditioned medium (ACM) has an effect on neurons. Astrocytes were treated with IL-1β and TNF-α for seven days; the treatment was then washed out, and the ACM was collected 48 h later. At this stage, the astrocytes still secreted a broad profile of inflammatory factors (Supplementary Fig. S5b). In-house-differentiated hPSC-derived neurons were exposed to ACM for 48 h, after which they still retained a normal branched neuronal morphology (Fig. 5a). Additionally, ACM treatment did not cause any changes in viability or apoptosis or cause cytotoxicity, confirming that the produced reactive astrocytes did not secrete neurotoxic factors at detrimental levels (Fig. 5b). Similar results were obtained with commercial hiPSC-derived neurons (Supplementary Fig. S6a–b).

The effect of ACM on neuronal functionality was studied with a microelectrode array (MEA) setup, which allowed repeated measurements from the same network. In-house-differentiated hPSC-derived neuronal cells were first cultured on MEAs for five weeks until mature bursting and synchronic activity was achieved throughout the network (Fig. 5c–d). Once array-wide activity had developed, the cultures were treated with control ACM or reactive ACM, and measurements were performed immediately (acute) and at 1 h, 4 h and 24 h after ACM application (Fig. 5e). A naïve control group received the same volume of intact medium (held for 48 h in an incubator similar to that in which the collected ACM was held). Overall, addition of the media induced increases in spike rates in all studied groups and, to a lesser extent, increases in burst counts during the 24 h follow-up period (Fig. 5e–f, Supplementary Table 2). The magnitudes of these changes were not as great in the commercial neurons as in the in-house-differentiated neurons (Supplementary Fig. S6d–e).

Next, activity changes in relation to baseline measurements were studied between the different treatment groups (Fig. 5g–h). The control ACM- and reactive ACM-treated networks responded similarly, as no significant differences between them were detected in spike or burst rates (Fig. 5g–h). This result was confirmed with both in-house-produced (Fig. 5g–h) and commercial neurons (Supplementary Fig. S6f–g).

In comparison to the control group, the reactive ACM group demonstrated an immediate increase in spike activity (Ctrl -0.168 ± 0.039 vs. reactive ACM 0.025 ± 0.036, p = 0.033) and a decrease in activity at the 24 h time point (Ctrl 0.470 ± 0.066 vs. reactive ACM 0.250 ± 0.087, p = 0.045, Fig. 5g–h). However, the control ACM group exhibited both increased spike activity (Ctrl 0.349 ± 0.067 vs. Ctrl ACM 0.576 ± 0.095, p = 0.039) and increased burst rate (Ctrl 0.579 ± 0.377 vs. Ctrl ACM 2.048 ± 0.535, p = 0.02) at the 1 h time point compared to the control group. Similar changes in spike and burst rates were also confirmed with commercial neurons (Supplementary Fig. S6f–g). Thus, conditioned medium from reactive astrocytes did not cause any detectable cytotoxicity or functional silencing of neuronal networks. In fact, both control and reactive ACM supported functional activity, suggesting that astrocytes were neurosupportive rather than neurotoxic under these conditions.

### Generation of a controlled astrocyte-neuron co-culture system utilizing a microfluidic device

**Figure 6:**
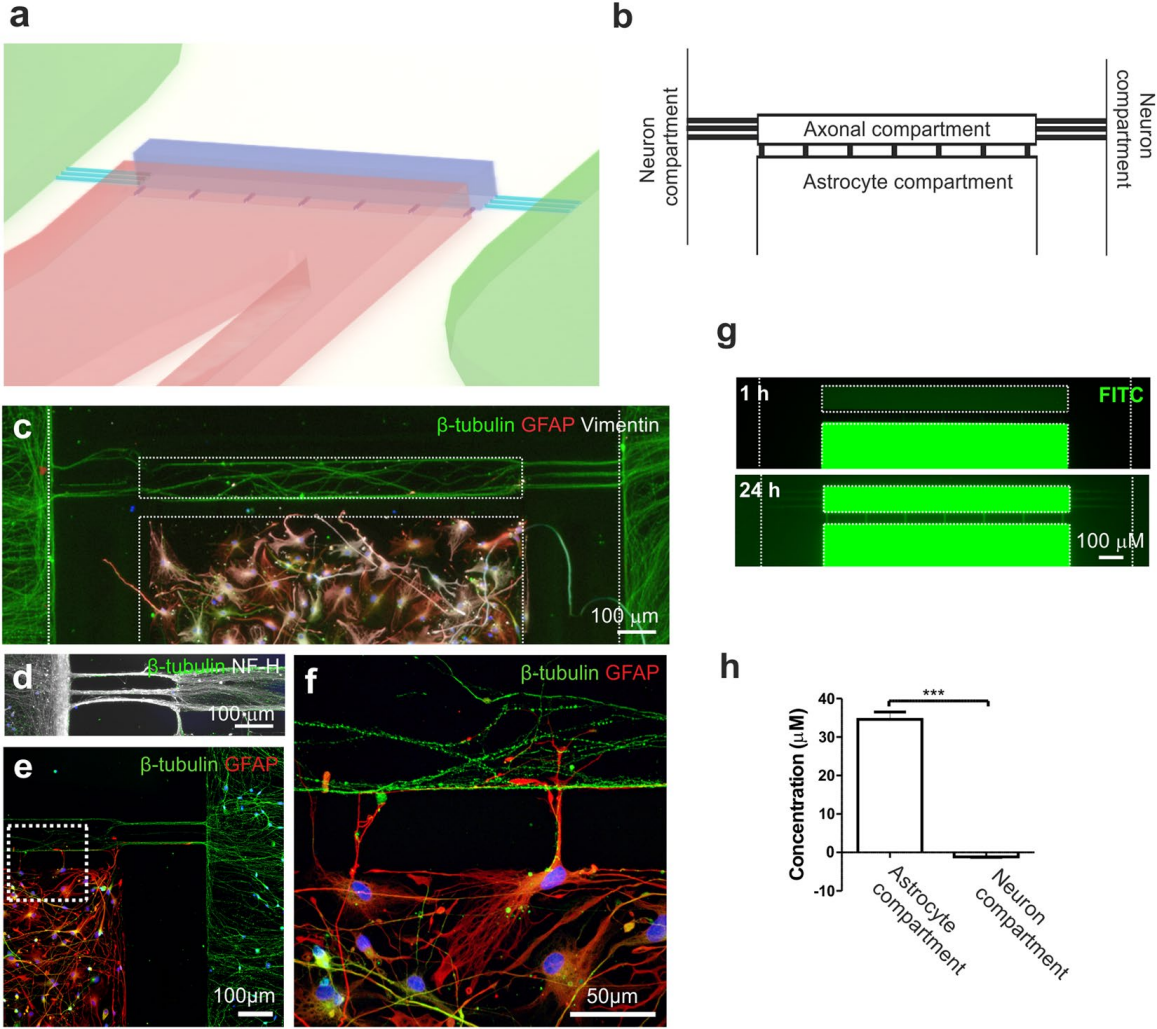
Description of the microfluidic device for co-culture of neurons and astrocytes and the fluidic isolation of the cell compartments. (**a**) Schematic showing a 3D illustration of the microfluidic device. (**b**) The microfluidic device consisted of two opposing compartments for neurons and a separate compartment for astrocytes, all of which were connected to an axonal compartment via microtunnels. (**c**) Immunocytochemical staining showing successful co-culture of β-tubulin-positive neurons and GFAP- and vimentin-positive astrocytes in the device and (**d**) the presence of NF-H-positive axons growing into the axonal compartment. (**e**) GFAP-positive astrocytes were placed close to the microtunnels leading to the axonal compartment, and (**f**) enlargement of the image illustrates cell-to-cell interactions between astrocytic processes and axons. (**g**) To evaluate the fluidic isolation of the astrocyte compartment, dextran-conjugated FITC was added to the astrocyte compartment, and its diffusion was evaluated by imaging with fluorescence microscopy at 1 h and 24 h. Fluorescence was detected in the axonal compartment after 24 h but was absent in the neuronal compartment (the compartments are shown with white dotted lines). (**h**) The concentration of dextran-FITC was measured after 24 h from medium collected from the astrocyte and neuron compartments. The data are shown as the mean ± s.e.m. (n = 6; the data are from one experiment). Statistical analysis was performed with independent-sample t-tests, and statistical significance is denoted as * p < 0.05, ** p < 0.01, and *** p < 0.001.

Next, we established a novel platform enabling controlled co-culture of neurons and astrocytes, targeted stimulation of astrocytes, and study of neuron-astrocyte interactions (Fig. 6a). The microfluidic device is composed of separate compartments for neurons and astrocytes, which are connected via microtunnels and axonal compartment (Fig. 6a,b, Supplementary Fig. S7). Immunofluorescence staining demonstrated that β-tubulin-positive neuronal somas were restricted to their designated compartments (Fig. 6c–e), while NF-H-positive axons grew readily through the microtunnels into the axonal compartment (Fig. 6d). GFAP-positive astrocytes were restricted to the astrocyte compartment (Fig. 6c), and direct cell-to-cell interactions could be verified, as astrocyte processes extended through the microtunnels and connected to neuronal axons in the neighboring compartment (Fig. 6e,f).

Fluidic isolation of the astrocyte compartment from the neuron compartment was demonstrated with FITC-conjugated dextran particles, which did not diffuse to the neuron compartment, as detected by fluorescence imaging (Fig. 6g) and measurements of particle concentrations after 24 h (Fig. 6h). However, over 24 h, particles did diffuse from the astrocyte compartment into the axonal compartment, verifying the possibility of studying humoral effects on axons with this system (Fig. 6g). In summary, the generated platform enabled the isolation of different cell types and cell parts, and the control of cell-to-cell interactions and fluid flow.

### Reactive astrocytes and an inflammatory environment affect axonal growth in the microfluidic platform

**Figure 7:**
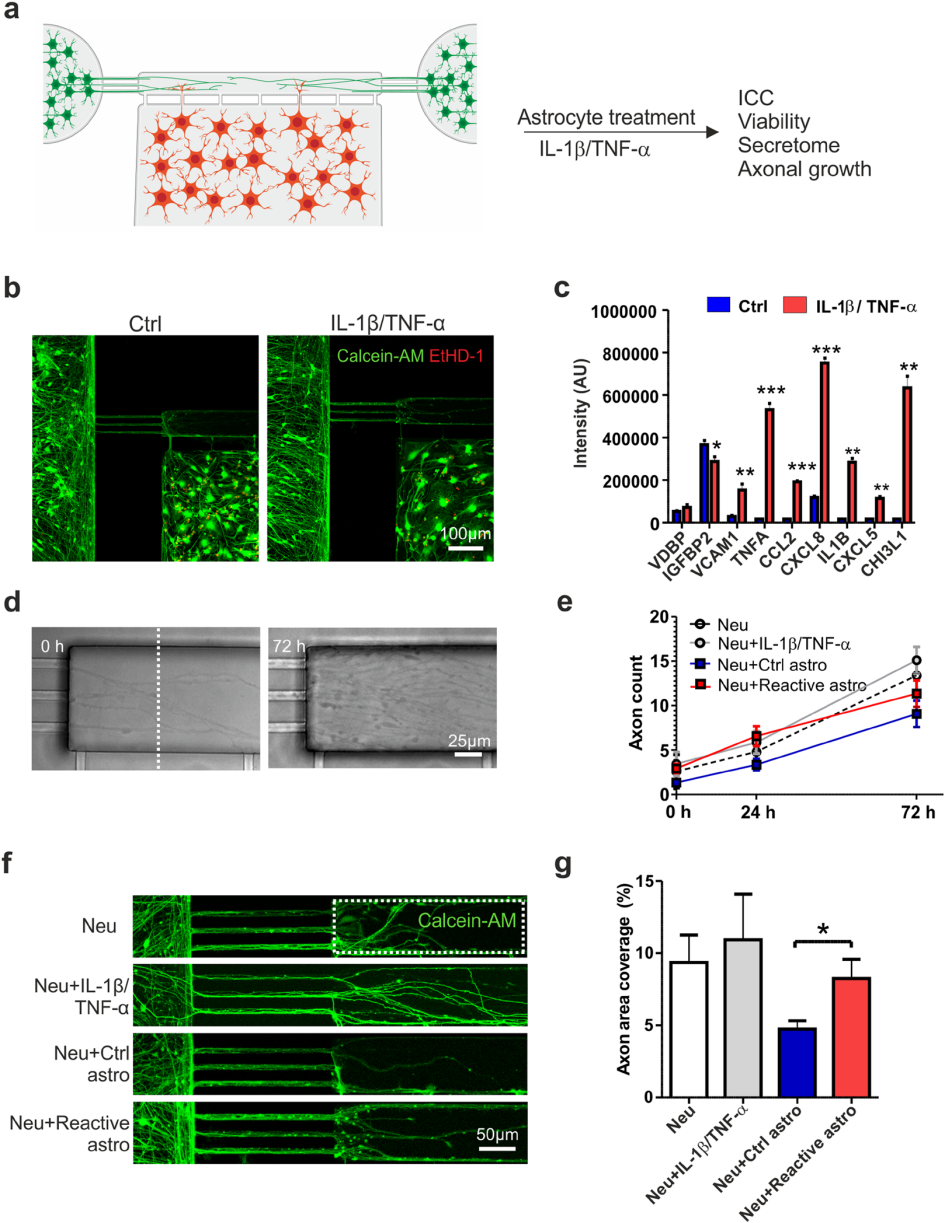
*In vitro* model for astrogliosis and axonal growth. (**a**) Experimental design for co-culture of astrocytes and neurons in the microfluidic device and astrocyte activation with IL-1β and TNF-α. (**b**) Both cell types remained viable (based on calcein-AM staining) on the platform after cytokine treatment, and very few dead cells (revealed by EtHD-1 staining) were observed. (**c**) Cytokine-treated astrocytes secreted significantly higher levels of several inflammatory factors than controls (n = 2; the data are from one experiment). (**d**) Phase-contrast images showing axons extending into the axonal compartment at 0 h and 72 h after cytokine treatment. (**e**) The number of axons extending into the axonal compartment (crossing the distance marked with the white dotted line) was quantified over 72 h. In addition to devices with co-cultures of control or reactive astrocytes and neurons (the Neu + Ctrl astro and Neu + Reactive astro groups), devices with cultures of only neurons were established to determine the effects of IL-1β and TNF-α treatment on axons (the Neu and Neu + IL-1β/TNF-α group) (n = 4–8 for each group; the data are from two experiments). (**f**) Axonal growth was also visualized with live-cell calcein-AM color staining to estimate coverage in the axonal compartment (the white dotted line). (**g**) Quantification of the area coverage in the axonal compartment revealed that reactive astrocytes supported axonal growth compared to control astrocytes (n = 6–13 for each group; the data are from one independent experiment). All data are presented as the mean ± s.e.m. Statistical analysis between groups was performed with independent-sample t-tests. P-values less than 0.05 were considered to indicate significance, and significant differences compared to the control group are presented in the graphs as * p < 0.05, ** p < 0.01, and *** p < 0.001.

The last aim was to induce the reactive astrocyte phenotype in the microfluidic platform and study the effect of reactive astrocytes on axon growth in the axonal compartment (Fig. 7a). To treat astrocytes, IL-1β and TNF-α were applied to the astrocyte compartment after the neurons and astrocytes were both plated into the microfluidic platform (Fig. 7a). The 72 h cytokine treatment did not cause any major cytotoxic effects on either cell type, as only a few dead cells were detected among viable cells (Fig. 7b). Similar to the findings under open-well conditions described above, the reactive astrocytes secreted a wide range of inflammatory factors (e.g., VCAM1, CCL2, CXCL8, CXCL5, and CHI3L1) (Fig. 7c, Supplementary Fig. S8).

After confirming the proinflammatory phenotype of the astrocytes, the effect of these astrocytes on axonal growth was studied by quantifying the number and density of axons extending to the axonal compartment (Fig. 7d–g). In addition to devices with co-cultures of control or reactive astrocytes with neurons (treatment groups: Neu + Ctrl astro and Neu + Reactive astro), devices with only neurons were included in the study to elucidate whether treatment with IL-1β and TNF-α alone had an effect on axons (treatment groups: Neu and Neu + IL-1β/TNF-α). Over the 72 h follow-up period, no statistically significant differences were detected in axon counts between the groups (Fig. 7e). Furthermore, axonal density was assessed with live-cell calcein-AM color staining by quantifying the area coverage in the axonal compartment 72 h after cytokine treatment (Fig. 7f,g). The results showed that reactive astrocytes increased axonal density compared to control astrocytes (Neu + Ctrl astro 4.7 ± 0.6 vs. Neu + Reactive astro 8.2 ± 1.3, p = 0.028, Fig. 7g). Taken together, the results indicate that selective cytokine stimulation of astrocytes resulted in an inflammatory environment that had a positive effect on axonal growth, demonstrating the suitability of the developed cell model for studying axonal biology.

## Discussion

Astrocytes are an acknowledged partner in the inflammatory processes in many neurodegenerative diseases and injuries of the CNS^5,11^. Astrocytes regulate the homeostasis of their surroundings and can have direct effects on neurons, both beneficial and detrimental. Human astrocytes are known for their complexity and heterogeneity, and some of their structural and functional traits differ from those of rodent astrocytes^12–14^. Therefore, in this study, we used hiPSC-derived astrocytes to create a human cell model system to study the inflammatory activation of astrocytes and the effects of activated astrocytes on neuronal functionality and axonal growth.

A variety of inflammatory molecules have been studied for their ability to induce a reactive astrocyte phenotype *in vitro*^8,23,43^. Here, astrocytes were treated with a combination of the cytokines IL-1β and TNF-α, which are typically released by activated microglia during neuroinflammation and are capable of stimulating astrogliosis *in vivo*^5,44,45^ and *in vitro*^18,46–48^. Furthermore, co-stimulation with IL-1β and TNF-α has been demonstrated to be a more potent trigger of reactivity that stimulation with either of the cytokines independently^8,23^. To date, few reports have described the responses of hiPSC-derived astrocytes to cytokine treatments^18,22–25,41^. However, many astrocyte differentiation protocols use serum to enhance astrocytes production that can promote astrocyte reactivation^14,23^. Here, astrocytes were cultured in serum-free medium, and under control conditions, the astrocytes remained in a quiescent state according to the results of gene expression and secretion profile analysis. We also treated astrocytes with the inflammatory cytokines for 7 days as described previously^18^ to mimic chronic effects. During the 7-day activation, we observed a gradual change in the morphology of the astrocytes from fibrous to polygonal and a reorganization of intermediate filament proteins, which are typical hallmarks of astrogliosis *in vivo*^6^. This clear morphological change has previously been demonstrated *in vitro* with human fetal astrocytes^14,46^ but not reported with hiPSC-derived astrocytes^18,22,25^. Additionally, upregulation of GFAP has been commonly described in relation to astrogliosis *in vivo*^10^. Interestingly, even though both control and reactive astrocytes stained positive for GFAP, more detailed analysis revealed downregulation of GFAP at both the gene and total protein levels upon cytokine treatment. Consistent with our data, reductions in GFAP have been reported in IL-1-treated human fetal astrocytes^49^ and in IL-1β- and TNF-α-treated rodent astrocytes at both the gene and protein levels^8,43^. Overall, we showed morphological transformation and alterations in the structural proteins of reactive astrocytes following 7-day IL-1β and TNF-α treatment.

Astrocytes are immunocompetent cells, and inflammatory activity upon stimulation is therefore their important feature^6^. The key signaling pathways involved in astrocyte reactivation include NF-κB pathway, which was acutely activated after cytokine treatment in the present study. NF-κB activation is known to induce the production of proinflammatory cytokines, chemokines and adhesion molecules, which mediate leukocyte recruitment to the CNS, leading to neuroinflammation^7,10,50^. Here, we demonstrated the secretion of a wide variety of chemokines, cytokines and other inflammatory factors by reactive astrocytes. For example, secretion of the typical inflammatory markers CCL5, CXCL8 and IL-6 was temporally induced after cytokine treatment, which has also been previously reported in studies on IL-1β- and TNF-α-treated hiPSC-derived astrocytes^18,22^. Interestingly, we observed upregulation of complement component 3 (C3), which has been linked to the neuroinflammatory phenotype of reactive astrocytes and has also been detected in demyelinating lesions of MS patients^8,9^. For the first time, we detected CHI3L1, OPN, and PTX3, known astrogliosis-associated markers *in vivo*^9^, in hiPSC-derived reactive astrocytes; these proteins are also considered candidate diagnostic and prognostic biomarkers for CNS diseases^51,52^. Thus, a broad range of inflammatory factors were discovered in a human model system of astrogliosis; however, the specific roles of these factors in neuroinflammatory processes remain to be explained.

Only a handful of studies have addressed interactions between reactive astrocytes and neurons *in vitro* using human cells^8,24,26,53^. Here, humoral signaling by reactive astrocytes sustained neuronal viability, suggesting that the astrocyte secretion profile was not primarily neurotoxic. In contrast, previous studies have described decreased numbers of neurons in direct co-cultures of human neurons and IL-1β-stimulated astrocytes^24^, and the humoral effects of TNF-α, IL-1α and complement component 1q (C1q) -stimulated rodent astrocytes have been reported to be toxic to human neurons^8^. Here, responses were also studied on a more sensitive neuronal network functionality level^54^. ACM exposure did not silence neuronal network activity but rather supported functionality, suggesting the polarization of astrocytes into a neurosupportive state. Previous studies have shown that secreted factors as well as direct cell-to-cell contact with rodent astrocytes can increase the activity of networks on MEAs^55,56^. On the other hand, neurotoxic astrocytes have failed to promote synapse function^8^. Altogether, our results suggest that 7-day stimulation of hiPSC-derived astrocytes with cytokines results in an inflammatory phenotype that has a supportive rather than a harmful effect on neuronal cells.

To reveal the underlying mechanisms of CNS disorders, the complex cellular interactions between glial and neuronal cells should be investigated in more detail. Microfluidic technology can provide *in vitro* research tools that go beyond conventional cell culture methods with the aim of better modeling *in vivo* environments^31,32^. Here, our microfluidic platform was developed to co-culture the two cell types in a compartmentalized, controlled manner to specifically study axonal interactions with glial cells. Co-culture of human neurons and astrocytes was successful in the platform, which allowed direct contact between astrocyte processes and axons. Moreover, fluidic isolation enabled targeted stimulation of astrocytes in their compartment while also permitting humoral effects on neuronal axons extending into the axonal compartment. Activation of astrocytes with IL-1β and TNF-α was reproduced selectively in the microfluidic platform, and the inflammatory niche promoted axonal growth, further suggesting neurosupportive characteristics. This interpretation is supported by a recent report in which IL-1β mediated a potentially neuroprotective astrocyte phenotype expressing axonal permissive transcripts^57^. A microfluidic platform has been previously used for analysis of the neuroinflammatory mechanisms of astrocytes and microglia in the neurotoxic environment of Alzheimer’s disease^26^. Our results present a unique microfluidic device designed for studying the interactions of axons and glial cells and further validate the suitability of microfluidic platforms as tools for studying the complex interplay among multiple cell types in the contexts of neuroinflammation and neurodegeneration.

In conclusion, our results suggest that extended co-stimulation with IL-1β and TNF-α produces a reactive astrocyte phenotype with a broad inflammatory secretion profile that displays neurosupportive characteristics with regard to neuronal viability, functionality and axonal growth. The mechanism behind the neurosupportive effects need to be clarified in the future studies. Furthermore, an *in vitro* model combining human cells and microfluidic technology was developed, and the usefulness of the proposed novel microfluidic platform for investigation of neuron-astrocyte interactions in an inflammatory environment was demonstrated. Advancing the field of *in vitro* modeling and creating tools for studying intricate cellular interactions in neurological disorders will promote the discovery of novel neuroprotective therapeutic targets in the future.

## Methods

### Human pluripotent stem cells and differentiation of neurons and astrocytes

The hPSC lines Regea 08/023^58^ and 10212.EURCCs^59^ used in this study were derived at the Faculty of Medicine and Health Technology (MET), Tampere University, Finland. MET has approval from the Finnish Medicines Agency (FIMEA) for research utilizing human embryos (Dnro 1426/32/300/05) and has received supportive statements from the regional ethics committee of Pirkanmaa Hospital District for the derivation, culture, and differentiation of hESCs (R05116) and hiPSCs (R08070). An informed consent was obtained from all subjects who provided cell samples. All methods were carried out in accordance with relevant guidelines and regulations. The hiPSC line 10212.EURCCs was derived from human skin fibroblasts using Sendai virus technology^60^. The hPSC lines were expanded in feeder-free culture as described earlier^61^. The in-house-produced neurons were differentiated according to the methods in a previous publication^62^, with some modifications (details in the Supplementary methods).

Commercial hiPSC-derived neurons (ax0018) and astrocyte progenitors (ax0083) from healthy donors were cultured according to the manufacturer’s protocol (both from Axol Bioscience Inc., UK). The astrocyte progenitors were differentiated for 24 days in culture and then treated for 7 days with 10 ng/ml human recombinant TNF-α and 10 ng/ml human recombinant IL-1β (PeproTech Inc.). Samples for astrocyte characterization were collected during the treatment at the 0 h, 24 h, 72 h and 7 day time points (Fig. 2a).

### RNA isolation and quantitative PCR

RNA was isolated from astrocytes with a NucleoSpin RNA kit (Macherey-Nagel). The concentration and purity of RNA were quantified with a NanoDrop 1000 (Thermo Fisher Scientific). RNA was converted to cDNA using a High Capacity cDNA Reverse Transcription Kit (Thermo Fisher Scientific). The expression levels of *CCL5, CXCL8, GFAP, LCN2, C3, GFAP, VIM, S100B, IL1R, IL1RAP, TNFRSF1A* and *TNFRSF1B* (Supplementary Table 3) were analyzed with TaqMan assays using an ABI Prism 7300 real-time PCR system (Thermo Fisher Scientific). The data were analyzed using the delta Ct method using GUSB and GAPDH as endogenous controls.

### Immunocytochemical staining

Immunocytochemical staining was performed according to the methods in a previous publication^63^. The used primary and secondary antibodies are listed in the Supplementary Table 4. Images were acquired with an Olympus IX51 microscope equipped with an Olympus DP30BW camera (Olympus Corporation, Germany) or an LSM780 laser-scanning confocal microscope equipped with a Quasar spectral GaAsP detector (all from Carl Zeiss, Germany). CellProfiler^64^ and CellProfiler Analyst^65^ software was used to perform image analysis. For evaluation of astrocyte morphology, the FormFactor module of CellProfiler software was used to calculate circularity index according to the area and perimeter metrics of each cell. Circularity index was calculated as 4*π*Area/Perimeter^2^, with a value of 1 describing a perfectly circular object.

### Glutamate uptake

Glutamate uptake by cultured cells was analyzed with a glutamate assay kit (Abcam). The cells were washed with PBS and with 0.1% BSA in PBS for 30 min + 37 °C. The cells were then incubated with 100 μM glutamate in PBS for 1 h + 37 °C. Finally, the cells were washed with PBS and lysed with assay buffer. The concentration of glutamate in the cell lysate was analyzed according to the manufacturer’s protocol.

### Cell proliferation assay

A CyQUANT Cell Proliferation Assay (Thermo Fisher Scientific) was used to analyze the DNA amounts in cultured cells according to the manufacturer’s protocol. Fluorescence was measured at 520 nm (Wallac Victor 1420, PerkinElmer).

### Cell viability and apoptosis assay

Cell viability and caspase-3/7 activation were detected in cultured cells with an ApoTox-Glo Triplex Assay (Promega). For positive controls, cells were treated with 1.25 µM staurosporine and 25 µM ionomycin (both from Sigma) for 6 h. Fluorescence was measured at 505 nm and 520 nm for viability and cytotoxicity, respectively. Luminescence was measured to detect apoptosis (Wallac Victor 1420).

### Collection of astrocyte conditioned medium

Astrocyte conditioned medium (ACM) from cytokine-treated and control astrocytes was collected at the 9-day time point. First, astrocytes were treated for 7 days with the cytokines IL-1β and TNF-α; thereafter, the cells were washed with PBS, and fresh medium was added to the cells. At day 9, the medium was harvested and centrifuged at 400 × *g* for 5 min at RT, and the supernatant was collected and stored at -80 °C until use. Neuronal cells (in-house-derived hESC line Regea 08/023) were exposed to ACM or control medium for 24 h (MEA analysis) or 48 h (viability assay) before analysis. The experiments with ACM also included a control group; the control medium was prepared similarly to the ACM but without astrocytes.

### Cytokine array and ELISA

Protein secretion (see the list of analytes in Supplementary Table 1) into the astrocyte culture medium was measured using a Proteome Profiler Antibody Array (Human XL Cytokine Array Kit, ARY022B, R&D Systems) according to the manufacturer’s protocol. The membranes were imaged with a ChemiDoc imaging system (Bio-Rad), and the intensity of the spots was quantified with Image Lab software (Bio-Rad).

The levels of IL-6 in the medium were measured with a human IL-6 uncoated ELISA kit (#88-7066-22, Thermo Fisher Scientific) according to the manufacturer’s protocol. The absorbance was measured at 450 nm (Wallac Victor 1420).

### Viability staining

A viability/cytotoxicity kit for mammalian cells (Thermo Fisher Scientific) was used to evaluate the viability of cells after ACM treatment and culture in the microfluidic platform. The cultures were incubated for 30 min to 1 h at 37 °C with green-fluorescent calcein-AM (0.5 µM) for detection of live cells and with red-fluorescent ethidium homodimer-1 (0.5 µM) for detection of dead cells. The samples were imaged immediately with an Olympus IX51 microscope equipped with an Olympus DP30BW camera (Olympus Corporation) and an LSM780 laser-scanning confocal microscope equipped with a Quasar spectral GaAsP detector (Carl Zeiss).

### Microelectrode array measurements

Neuronal network activity was recorded with an Axion Maestro system controlled by AxIS Software (Axion Biosystems, Atlanta, GA, USA) with 12.5 kHz sampling rate as described previously^66^. The hPSC-derived cortical neurons (in-house-derived hESC line Regea 08/023 or commercial hiPSC-derived neurons, ax0018) were plated on CytoView MEA 48 plates with 16 electrodes per array. Recordings were obtained under a controlled temperature of 37 °C. Spontaneous activity development was examined once a week with 10-minute recordings. After five weeks, when synchronous network activity had developed, the experiments with astrocyte conditioned medium (ACM) were initiated. The responses to control medium, control ACM or reactive ACM were measured for 10 min immediately and at 1 h, 4 h and 24 h after application. Analysis methods for spike and burst detection are described in the Supplementary methods section.

### Design of the microfluidics device

An in-house-developed microfluidics polydimethylsiloxane (PDMS) device (Patent: WO2015092141A1) was used (Fig. 6a, Supplementary Fig. S7). The device contains three separate cell compartments and an axonal compartment that are interconnected by microtunnels (Fig. 6b, Supplementary Fig. S7). There are two neuronal compartments (length = 4250 µm, width = 3000 µm) that are connected to the axonal compartment (length = 1000 µm, height = 100 µm, width = 100 µm) with three microtunnels (length = 250 µm, height = 3 µm, width = 10 µm). The microtunnels allow the extension of axons but prevent the migration of neuronal somas from the neuronal compartment into the axonal compartment (Fig. 6b,c). The flow-based astrocyte compartment is connected to the axonal compartment with seven microtunnels (length = 50 µm, height = 3.5 µm, width = 10 µm) (Supplementary Fig. S7). The fabrication of PDMS devices are described in the Supplementary methods.

### Co-culture of neurons and astrocytes in the microfluidic platform

HiPSC-derived cortical neurons (10212.EURCCs) and commercial hiPSC-derived astrocytes (ax0083) were seeded into the neuronal compartments and astrocyte compartment, respectively (see details on Supplementary methods). Three days after cell plating, 10 ng/ml IL-1β- and TNF-α-containing medium was added into the astrocyte compartment, and treatment continued for 3 days. After cytokine treatment, the viability of cells was assessed with viability staining, and astrocyte-secreted inflammatory factors were analyzed in medium collected from the astrocyte compartment. To characterize the astrocytes and neurons in the co-cultures, immunofluorescence staining was performed. First, the microfluidics devices were separated manually from the coverslips, and then the cells were fixed with 4% PFA and stained according to an immunocytochemical protocol. To evaluate the influences of the astrocytes and cytokines on axonal growth, the following experimental groups were established: 1) a group in which neurons were seeded in the neuronal compartments (the Neu group), 2) a group in which neurons were seeded in the neuronal compartments and cytokine treatment was conducted via the astrocyte compartment (the Neu + IL-1β/TNF-α group), 3) a group in which neurons and astrocytes were seeded in their compartments (the Neu + Ctrl astro group), and 4) a group in which neurons and astrocytes were seeded in their compartments and cytokine treatment was conducted via the astrocyte compartment (the Neu + Reactive astro group). The cells in the devices were imaged with phase-contrast microscopy at baseline and at 24 h and 72 h after cytokine treatment. The individual axons entering the axonal compartment were counted manually from the images at a distance of 80 µm from the axonal microtunnels (the white dotted line in Fig. 7d). The density of the axons in the axonal compartment was evaluated by analyzing the area covered with calcein-AM-stained axons at 72 h after cytokine treatment. The analyzed area was a 0.033 mm^2^ (325 µm × 100 µm) region next to the axonal microtunnels (the white dotted line in Fig. 7f), and analysis was performed using CellProfiler software.

### Statistical analysis

Statistical analysis of Gaussian-distributed data was performed with independent-sample T-tests. If the data followed a non-Gaussian distribution, the nonparametric Mann-Whitney U-test was used. Bonferroni correction was used for multiple comparisons.

The MEA data followed a non-Gaussian distribution; therefore, nonparametric tests were selected. To study changes in activity within treatment groups over time, the Friedman test for related samples was performed followed by the Wilcoxon signed-rank test to compare two related samples. To study differences between groups, delta values were calculated (by subtracting the baseline values from the values obtained immediately (acute), 1 h, 4 h, or 24 h after treatment), and Mann-Whitney U-tests were performed.

A p-value < 0.05 was considered to indicate significance. Statistical significance is denoted as * p < 0.05, ** p < 0.01, and *** p < 0.001. All statistical tests were performed with SPSS Statistics software (version 25.0).

## Supplementary information

Supplementary information

## Data Availability

The datasets generated during and/or analyzed during the current study are available from the corresponding author on reasonable request.
