# Supplementary material for: Co-stimulation with IL-1β and TNF-α induces an inflammatory reactive astrocyte phenotype with neurosupportive characteristics in a human pluripotent stem cell model system
Source: Sci Rep. 2019 Nov 15;9:16944. doi: 10.1038/s41598-019-53414-9 (PMC6858358; doi:10.1038/s41598-019-53414-9)
Supplement: Supplementary file 1 — Supplementary information [file 41598_2019_53414_MOESM1_ESM.pdf]

Supplementary information

Co-stimulation with IL-1β and TNF-α induces an inflammatory reactive astrocyte phenotype with neurosupportive characteristics in a human pluripotent stem cell model system

T. Hyvärinen [#], S. Hagman[#], M. Ristola, L. Sukki, K. Veijula, J. Kreutzer, P. Kallio, S. Narkilahti

# These authors contributed equally to this work

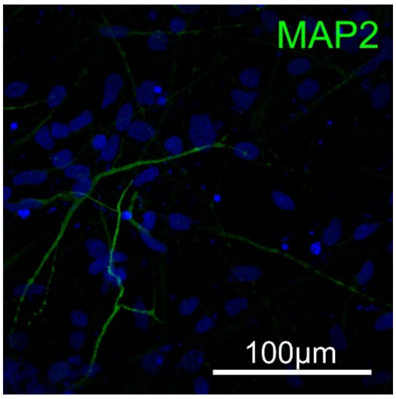

Supplementary Fig. S1. Immunocytochemical characterization of hiPSC-derived astrocytes. The astrocyte progenitor population contained only a few contaminating MAP2-positive neuronal cells.

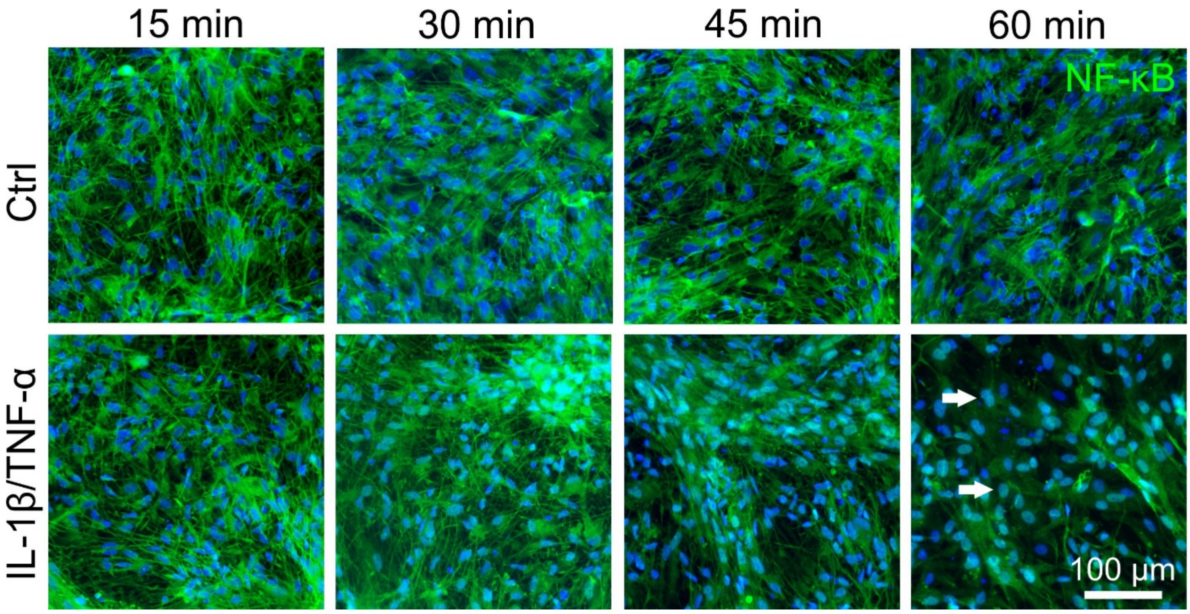

Supplementary Fig. S2. Activation of the NF-κB pathway in hiPSC-derived astrocytes after IL-1β and TNF-α treatment. In control astrocytes, NF-κB was ubiquitously expressed in the cytoplasm, while in cytokine-treated astrocytes, activation and translocation of NF-κB to the nucleus (white arrows) occurred rapidly in minutes.

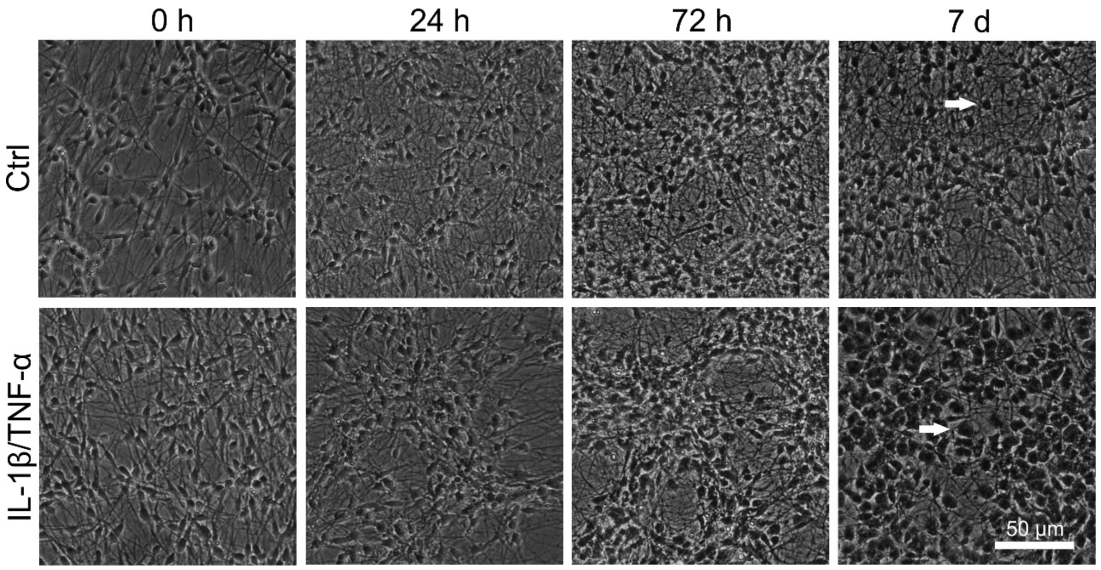

Supplementary Fig. S3. Morphological changes in hiPSC-derived astrocytes after IL-1β and TNF-α treatment. Astrocytes experienced a gradual morphological change from fibrous to flattened (white arrows) over the 7-day period of cytokine treatment.

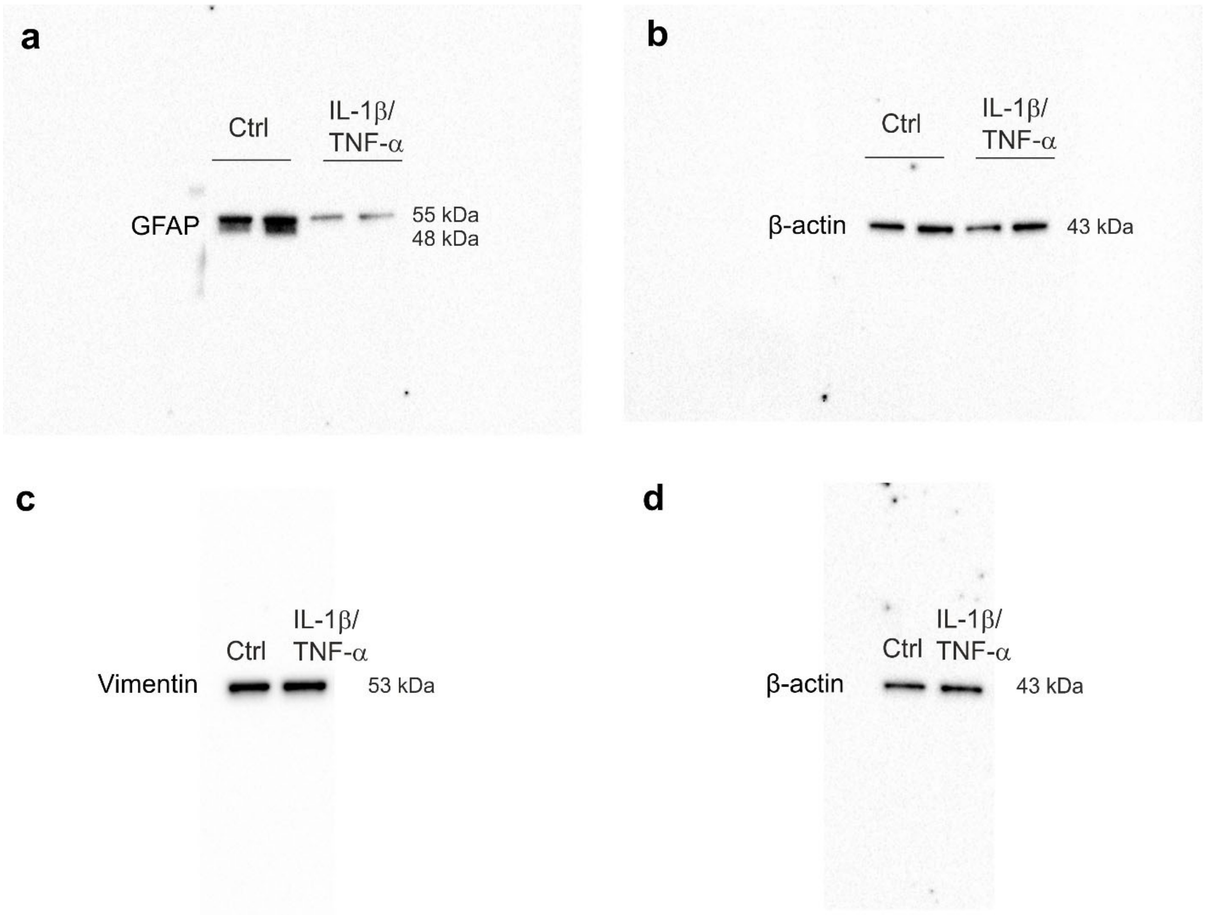

Supplementary Fig. S4. Full-length images of western blot membranes. (a) The GFAP protein levels in control and IL-1β- and TNF-α-treated astrocytes were analyzed by western blotting. **(b)** β-Actin was used as a loading control. **(c)** The vimentin protein levels in control and IL-1β- and TNF-α-treated astrocytes were analyzed by western blotting. **(d)** β-Actin was used as a loading control.

Supplementary Table 1. Proteins analyzed with the proteome array.

Analyte	Description
ACAN	Aggrecan
ADIPOQ	Adiponectin
AGER	Advanced Glycosylation End-Product Specific Receptor
ANG	Angiogenin
ANGPT1	Angiopoietin 1
ANGPT2	Angiopoietin 2
APOA1	Apolipoprotein A1
BDNF	Brain Derived Neurotrophic Factor
BSG	Basigin
C5	Complement component C5
CCL2	C-C Motif Chemokine Ligand 2
CCL3	C-C Motif Chemokine Ligand 3
CCL5	C-C Motif Chemokine Ligand 5
CCL7	C-C Motif Chemokine Ligand 7
CCL17	C-C Motif Chemokine Ligand 17
CCL19	C-C Motif Chemokine Ligand 19
CCL20	C-C Motif Chemokine Ligand 20
CXCL1	C-X-C Motif Chemokine Ligand 1
CXCL4	C-X-C Motif Chemokine Ligand 4
CXCL5	C-X-C Motif Chemokine Ligand 5
CXCL8	C-X-C Motif Chemokine Ligand 8
CXCL9	C-X-C Motif Chemokine Ligand 9
CXCL10	C-X-C Motif Chemokine Ligand 10
CXCL11	C-X-C Motif Chemokine Ligand 11
CXCL12	C-X-C Motif Chemokine Ligand 12
CD14	CD14 Molecule
CD40LG	CD40 ligand
CFD	Complement Factor D
CHI3L1	Chitinase 3 Like 1
CRP	C-Reactive Protein
CSF	Colony Stimulating Factor 1
CSF2	Colony Stimulating Factor 2
CSF3	Colony Stimulating Factor 3
CST3	Cystatin C
DKK1	Dickkopf WNT Signaling Pathway Inhibitor 1
DPP4	Dipeptidyl Peptidase 4
EGF	Epidermal Growth Factor
ENG	Endoglin
FASLG	Fas Ligand
FGF19	Fibroblast Growth Factor 19
FGF2	Fibroblast Growth Factor 2
FGF7	Fibroblast Growth Factor 7
FLT3	Fms Related Tyrosine Kinase 3
GDF15	Growth Differentiation Factor 15
GH	Growth Hormone 1
HGF	Hepatocyte Growth Factor
ICAM1	Intercellular Adhesion Molecule 1
IFNG	Interferon Gamma
IGFBP2	Insulin Like Growth Factor Binding Protein 2
IGFBP3	Insulin Like Growth Factor Binding Protein 3
IL1A	Interleukin 1 Alpha
IL1B	Interleukin 1 Beta

IL1RL1	Interleukin 1 Receptor Like 1
IL1RN	Interleukin 1 Receptor Antagonist
IL2	Interleukin 2
IL4	Interleukin 4
IL5	Interleukin 5
IL6	Interleukin 6
IL10	Interleukin 10
IL11	Interleukin 11
IL12p70	Interleukin 12 p70 subunit
IL13	Interleukin 13
IL15	Interleukin 15
IL16	Interleukin 16
IL17A	Interleukin 17A
IL18BP	Interleukin 18 Binding Protein
IL19	Interleukin 19
IL22	Interleukin 22
IL23	Interleukin 23
IL24	Interleukin 24
IL27	Interleukin 27
IL3	Interleukin 3
IL31	Interleukin 31
IL32	Interleukin 32
IL33	Interleukin 33
IL34	Interleukin 34
KLK3	Kallikrein Related Peptidase 3
LCN2	Lipocalin 2
LEP	Leptin
LIF	Leukemia inhibitory factor
MIF	Macrophage Migration Inhibitory Factor
MMP9	Matrix Metallopeptidase 9
MPO	Myeloperoxidase
OPN	Osteopontin
PDGFA	Platelet Derived Growth Factor Subunit A
PDGFB	Platelet Derived Growth Factor Subunit B
PLAUR	Plasminogen Activator, Urokinase Receptor
PTX3	Pentraxin 3
RBP4	Retinol Binding Protein 4
RETN	Resistin
RLN2	Relaxin 2
SERPINE1	Serpin Family E Member 1
SHBG	Sex Hormone Binding Globulin
TDGF1	Teratocarcinoma-Derived Growth Factor 1
TFF3	Trefoil Factor 3
TFRC	Transferrin Receptor
TGFA	Transforming Growth Factor Alpha
THBS1	Thrombospondin-1
TNFA	Tumor Necrosis Factor Alpha
TNFRSF8	TNF Receptor Superfamily Member 8
TNFSF13B	TNF Superfamily Member 13b
VCAM1	Vascular Cell Adhesion Molecule 1
VDBP	Vitamin D binding protein
VEGFA	Vascular Endothelial Growth Factor A

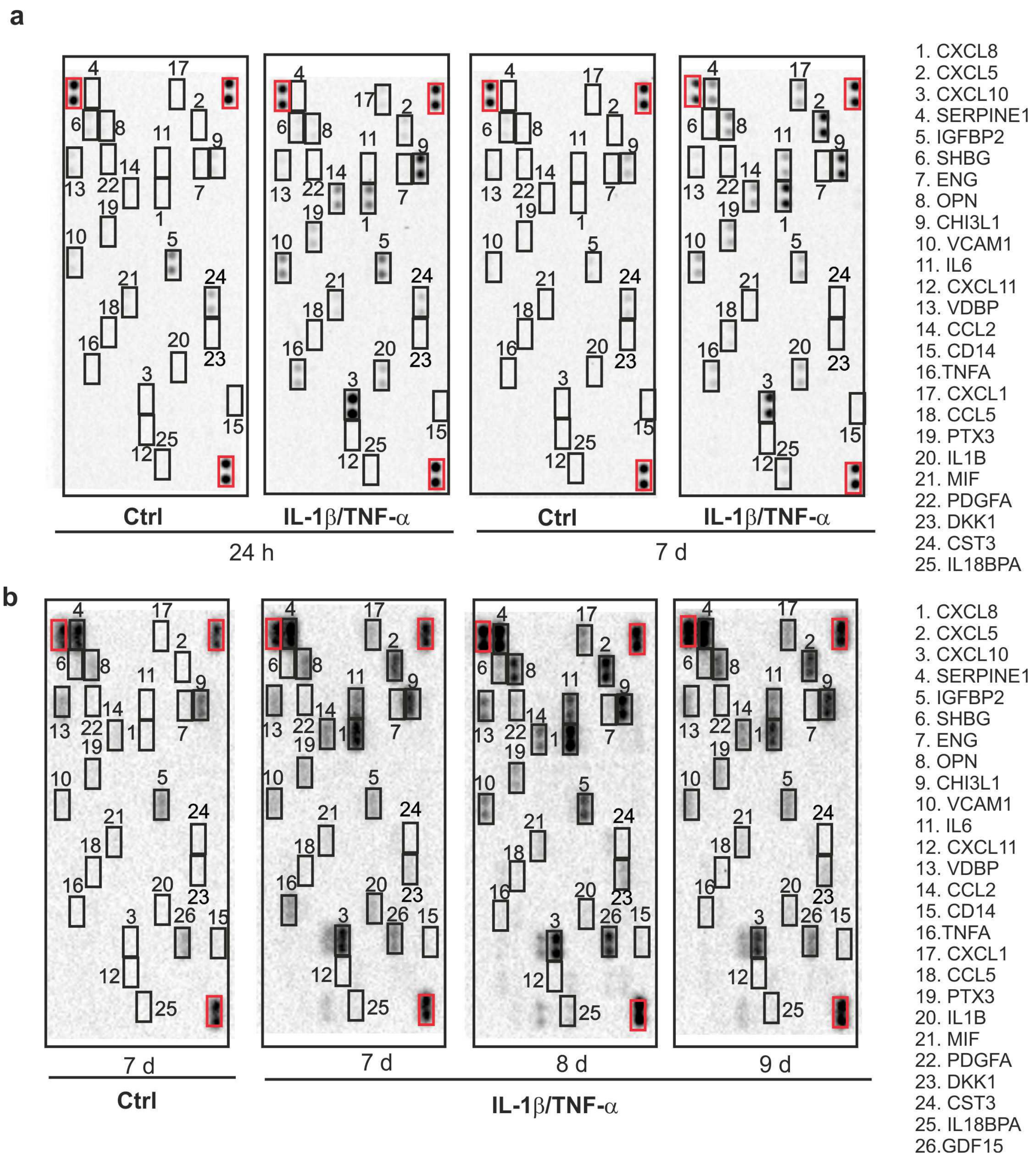

Supplementary Fig. S5. Proteome profile blots after cytokine treatments. (a) Astrocyte-secreted inflammatory factors were analyzed at the 24 h and 7-day time points for control astrocytes and reactive astrocytes. The blots are representative of the data from two independent experiments. **(b)** The secretion profiles of reactive astrocytes were confirmed at the 7-, 8- and 9-day time points. The astrocytes were first treated with IL-1β and TNF-α for 7 days; thereafter, the cytokines were removed from the astrocytes, which were cultured without cytokines for an additional 2 days (the 8 d and 9 d time points) before collection of astrocyte conditioned medium. The blots are from one experiment. The most upregulated proteins are marked with rectangles in the images, and the corresponding analytes are listed on the right. The red rectangles show the positive controls of the arrays.

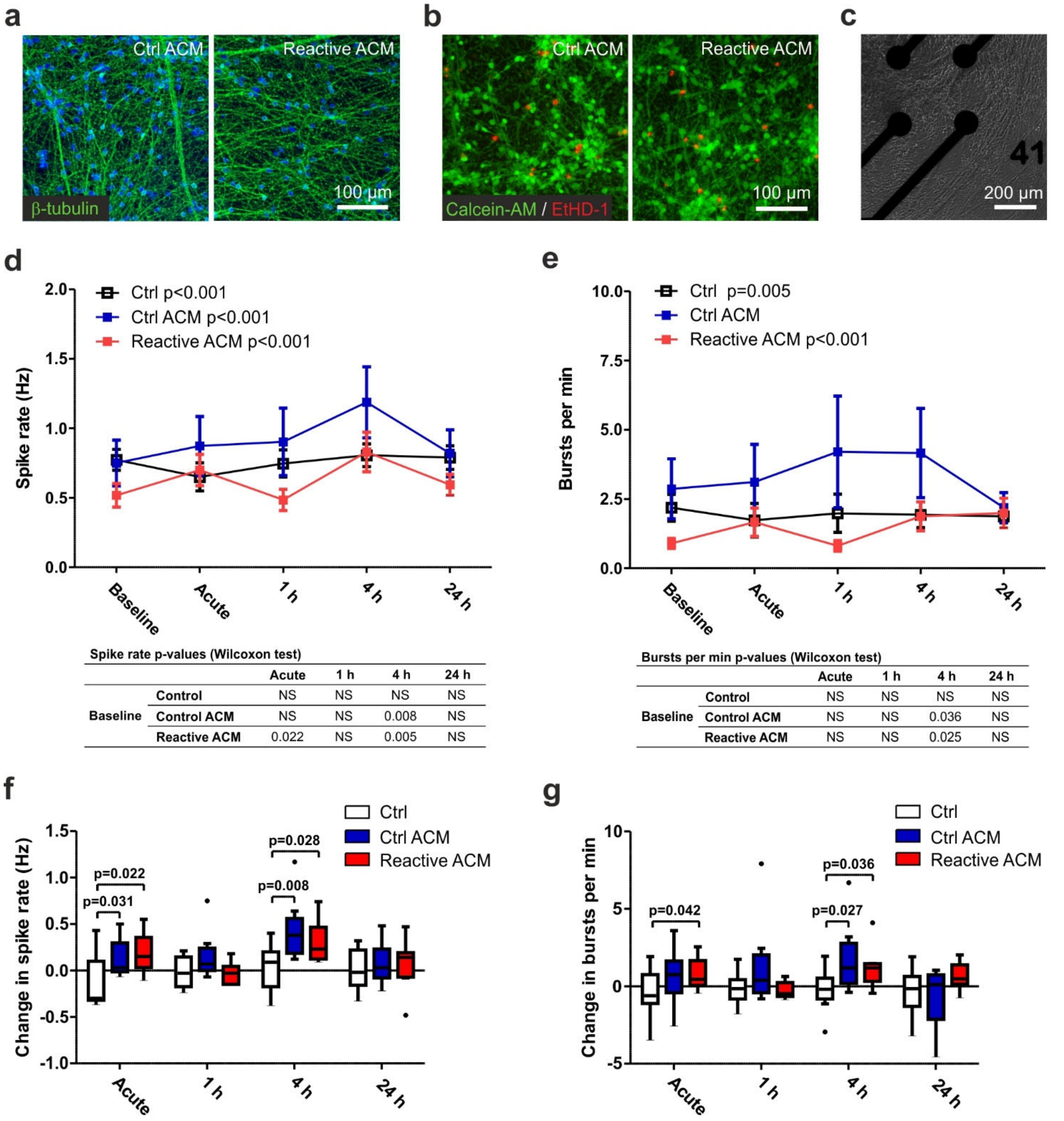

Spike rate p-values (Wilcoxon test)

		Acute	1 h	4 h	24 h
Baseline	Control	NS	NS	NS	NS
	Control ACM	NS	NS	0.008	NS
	Reactive ACM	0.022	NS	0.005	NS

Bursts per min p-values (Wilcoxon test)

		Acute	1 h	4 h	24 h
Baseline	Control	NS	NS	NS	NS
	Control ACM	NS	NS	0.036	NS
	Reactive ACM	NS	NS	0.025	NS

Supplementary Fig. S6. Viability and functionality of commercial hiPSC-derived neuronal cells after ACM treatment. (a) The commercial hiPSC-derived neuronal cells were positive for the neuronal marker β-tubulin and maintained axonal networks after 48 h of treatment with control ACM and reactive ACM. DAPI staining is shown in blue. **(b)** Live/dead staining showed that neuronal cells were viable after 48 h of treatment with control ACM and reactive ACM. Calcein-AM staining indicates live cells (green), and ethidium homodimer-1 (EtHD-1) staining indicates dead cells (red; n=3 cultures derived from one experiment). **(c)** The hiPSC-derived neuronal cells were cultured on MEAs for five weeks before the experiments to achieve mature functional activity. **(d)** Graph showing the development of spike rates over time in neuronal networks exposed to control medium, control ACM or reactive ACM. The table below shows the significant p-values for the spike rate changes over time within the treatment groups (NS=not significant). **(e)** Graph showing the development of burst rates over time in the control, control ACM and reactive ACM treatment groups. The table

below shows the p-values for the changes in the bursts per min over time within the treatment groups. The data are shown as the mean ± s.e.m. Statistical analysis within groups was performed with the Friedman test (shown in the images for d and e) followed by the Wilcoxon signed-rank test (shown in the tables for d and e). **(f-g)** To highlight significant differences between treatment groups, the data from panels d-e were reused and are presented as the changes in spike and burst rates compared to the baseline measurements. The data in (f) and (g) are shown as Tukey boxplots, and statistical significance was tested with the Mann-Whitney U-test. For the MEA results, n=9-10 networks derived from one differentiation.

Supplementary Table 2. Spike and burst activity of hPSC-derived neurons after control ACM and reactive ACM treatment at different time points. N=12 networks derived from one differentiation. Statistical testing was conducted within treatment groups between baseline and the different time points (acute, 1 h, 4 h, and 24 h) using the Wilcoxon signed-rank test. $P < 0.05$ was considered to indicate statistical significance.

		**Spike rate (Hz)**			**Burst per min**		
		**Ctrl**	**Ctrl ACM**	**Reactive ACM**	**Ctrl**	**Ctrl ACM**	**Reactive ACM**
**Baseline**	**mean**	0.861	1.154	1.095	2.116	3.621	3.368
	**s.e.m.**	0.098	0.195	0.140	0.324	0.694	0.779
**Acute**	**mean**	0.693	1.119	1.070	1.521	3.659	2.595
	**s.e.m.**	0.081	0.229	0.157	0.363	0.822	0.582
	**p-value**	0.002	NS	NS	NS	NS	NS
**1 h**	**mean**	1.241	1.735	1.603	2.695	5.669	4.273
	**s.e.m.**	0.161	0.275	0.249	0.329	0.879	0.802
	**p-value**	0.002	0.002	0.002	NS	0.008	NS
**4 h**	**mean**	1.700	2.020	1.899	4.105	8.227	5.701
	**s.e.m.**	0.204	0.289	0.259	0.438	1.700	1.174
	**p-value**	0.002	0.002	0.002	0.003	0.002	0.002
**24 h**	**mean**	1.332	1.689	1.345	3.092	6.868	4.826
	**s.e.m.**	0.132	0.304	0.194	0.504	2.666	1.490
	**p-value**	0.002	0.003	0.019	NS	NS	NS

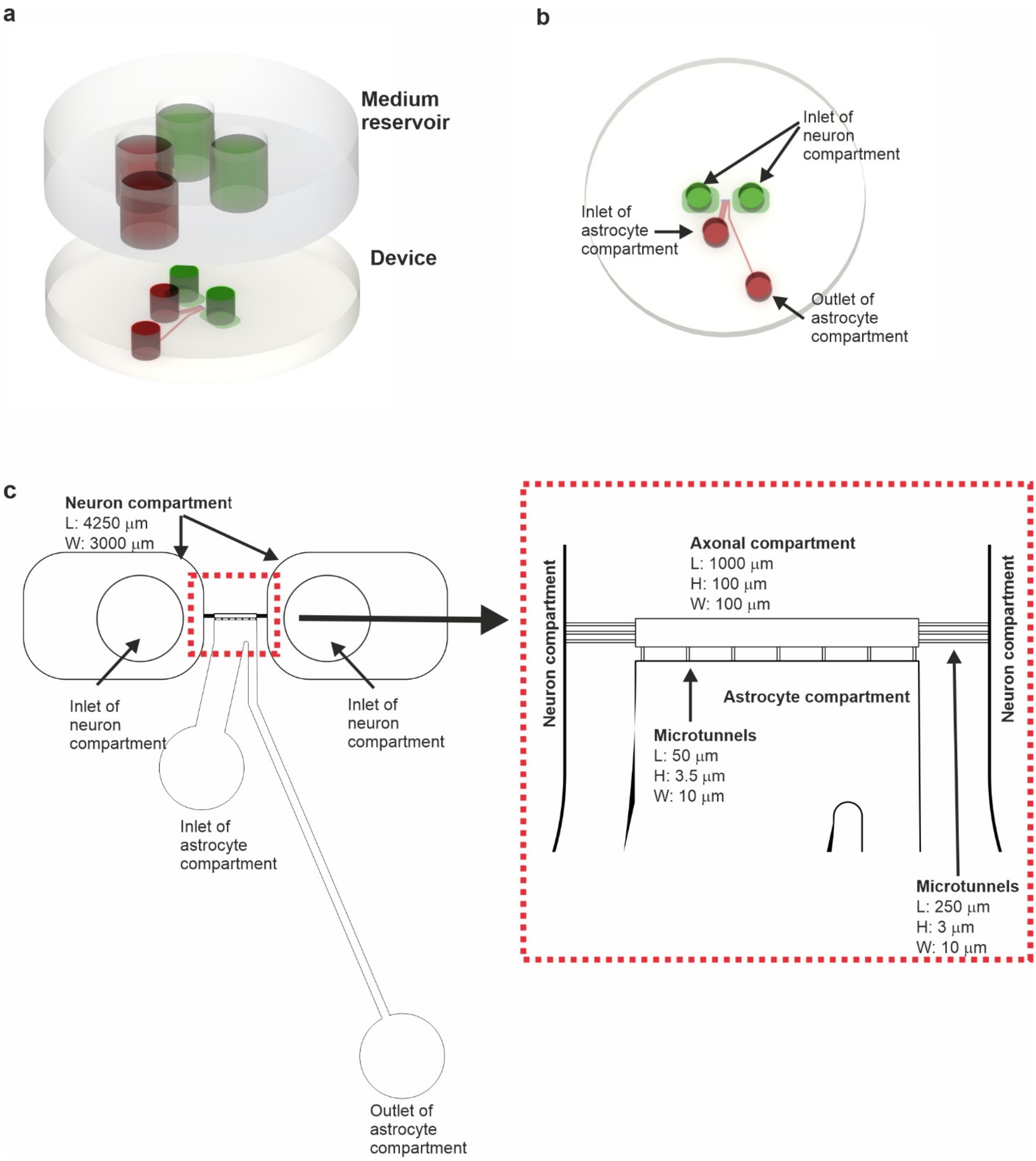

Supplementary Fig. S7. Design of the microfluidic platform. (a) Microfluidic platform is composed two separate PDMS parts: medium reservoir and microfluidic device containing three cell compartments connected via microtunnels. **(b)** The device contains three inlets and one outlet that connect the cell compartments to the medium reservoir and facilitate cell plating on their designated compartments. **(c)** The device consists of two large neuron compartments and a channel-like astrocyte compartment, all of which are connected to an axonal compartment via microtunnels. Enlargement describes the design and dimensions of the axonal compartment and its microtunnels (L=length; H=height, W=width).

Supplementary Table 3. Primer information for gene expression analysis.

Gene symbol	Assay ID	Gene name
C3	Hs00163811_m1	Complement component 3
CCL5	Hs00982282_m1	C-C motif chemokine ligand 5
CXCL8	Hs00174103_m1	C-X-C motif chemokine ligand 8
EAAT1	Hs00188193_m1	Excitatory amino acid transporter 1
EAAT2	Hs01102423_m1	Excitatory amino acid transporter 2
GFAP	Hs00909236_m1	Glial fibrillary acidic protein
IL1R1	Hs00991010_m1	Interleukin 1 receptor type 1
IL1RAP	Hs00895050_m1	Interleukin 1 receptor accessory protein
LCN2	Hs01008571_m1	Lipocalin 2
S100B	Hs00902901_m1	S100 calcium binding protein B
TNFRSF1A	Hs01042313_m1	TNF receptor superfamily member 1A
TNFRSF1B	Hs00961750_m1	TNF receptor superfamily member 1B
VIM	Hs00958111_m1	Vimentin
GAPDH	Hs02786624_g1	Glyceraldehyde-3-phosphate dehydrogenase
GUSB	Hs00939627_m1	Glucuronidase beta

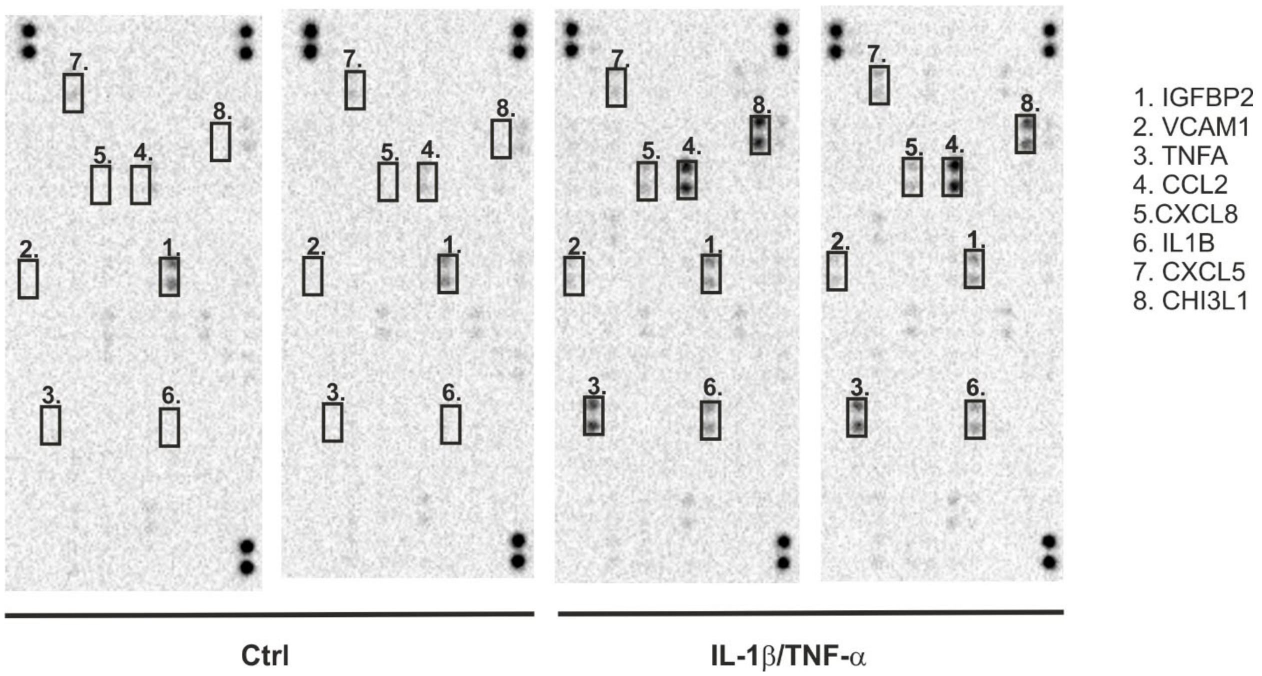

Supplementary Fig. S8. Secretion of proteins from reactive and control astrocytes during culture on the microfluidic device. Secretion of inflammatory factors by control astrocytes and reactive astrocytes was analyzed at 72 h. To analyze the factors secreted from astrocytes, medium was collected from the astrocyte compartment, and the inflammatory factor levels were analyzed with a cytokine array. The most upregulated proteins are marked with rectangles in the image, and the names of the analytes are listed on the right.

Supplementary Table 4. Primary and secondary antibodies used for the immunocytochemical stainings.

Primary antibodies

Protein name	Species	Working dilution	Supplier	Product number
ALDH1L1	rabbit	1:500	Abcam	ab87117
β_{III}tubulin	rabbit	1:2000	GenScript	A01627
EAAT1	rabbit	1:500	Abcam	ab416
EAAT2	mouse	1:500	Abcam	ab77039
GFAP	chicken	1:4000	Abcam	ab4674
GS	rabbit	1:500	Abcam	ab73593
Ki67	rabbit	1:800	Millipore	AB9260
MAP2	rabbit	1:400	Millipore,	AB5622
NF-H	chicken	1:500	GenScript	A00136
NF-κB p65	rabbit	1:400	Cell Signaling Technology	D14E12
S100β	mouse	1:500	Abcam	ab11178
vimentin	mouse	1:500	Dako	M0725

Secondary antibodies

Antibody	Working dilution	Supplier	Product number
Donkey anti-mouse Alexa Fluor 488	1:400	Thermo Fisher Scientific	A21202
Donkey anti-rabbit Alexa Fluor 488	1:400	Thermo Fisher Scientific	A21206
Donkey anti-mouse Alexa Fluor 568	1:400	Thermo Fisher Scientific	A10037
Donkey anti-rabbit Alexa Fluor 568	1:400	Thermo Fisher Scientific	A10042
Goat anti-chicken Alexa Fluor 568	1:400	Thermo Fisher Scientific	A11041
Goat anti-chicken Alexa Fluor 647	1:200	Thermo Fisher Scientific	A21449

Supplementary methods

Human pluripotent stem cells

The human embryonic stem cell (hESC) line Regea 08/023 (passage 6) [1] and the human induced pluripotent stem cell (hiPSC) line 10212.EURCCs (passage 6) were used in this study. The hPSC lines were derived at the Faculty of Medicine and Health Technology, Tampere University, Finland. The Faculty of Medicine and Health Technology has approval from the Finnish Medicines Agency (FIMEA) for research utilizing human embryos (Dnro 1426/32/300/05) and has received supportive statements from the regional ethics committee of Pirkanmaa Hospital District for the derivation, culture, and differentiation of hESCs (R05116) and hiPSCs (R08070). The hPSC lines were expanded in feeder-free culture on recombinant human laminin-521 (LN521, BioLamina) and E8 medium (Thermo Fisher Scientific) as described earlier [2]. These hPSCs were used for in-house neuronal cell differentiation. The pluripotency of the hPSC lines was controlled regularly, and all cultures maintained normal karyotypes and were mycoplasma free.

Differentiation of neurons and astrocytes

The in-house-produced neurons were differentiated according to the methods in a previous publication [3], with some modifications. Briefly, the basal medium was neural maintenance medium that consisted of 1:1 D-MEM/F12 with GlutaMAX and Neurobasal, 0.5% N2, 1% B27 with retinoic acid, 0.5 mM GlutaMAX, 0.5% NEEA, 50 µM 2-mercaptoethanol (all from Thermo Fisher Scientific), 2.5 µg/ml insulin (Sigma) and 0.1% penicillin/streptomycin (Thermo Fisher Scientific). Sequential coating with 100 µg/ml poly-L-ornithine (PO, Sigma) and 15 µg/ml LN521 was performed for plastic well plates and glass coverslips, and 0.1% polyethyleneimine (PEI, Sigma) and 50 µg/ml LN521 were used for MEAs. The cells were passaged with Accutase (Thermo Fisher Scientific) and plated in medium containing 10 µM ROCK Inhibitor (Sigma). For neural induction, the neural maintenance medium was supplemented with 100 nM LDN193189 and 10 µM SB431542 (both from

Sigma) for 12 days. Neural progenitor cells were expanded in maintenance medium supplemented with 20 ng/ml fibroblast growth factor-2 (FGF2, R&D Systems) until day 25. Final maturation was achieved by supplementing the maintenance medium with 20 ng/ml brain-derived neurotrophic factor (BDNF, R&D Systems), 10 ng/ml glial-derived neurotrophic factor (GDNF, R&D Systems), 500 µM dibutyryl-cyclic AMP (db-cAMP, Sigma) and 200 µM ascorbic acid (AA, Sigma). At day 32, the cells were plated for experiments. The cells were plated on plastic well plates at a density of 50000 cells/cm^2 except in the cell viability and apoptosis assays (100000 cells/cm^2) and on MEAs at a density of 1×10^6 cells/cm^2. The medium was changed every two to three days.

Western blot analysis

Cells were washed with ice-cold PBS, lysed in Laemmli sample buffer (Bio-Rad) and heated for 5 min at 95°C before storage at -80°C. The total protein concentrations were measured using a Pierce 660 nm Protein Assay (Thermo Fischer Scientific) with Ionic Detergent Compatibility Reagent (Thermo Fischer Scientific), and equal amounts of protein were loaded onto 10% Mini-PROTEAN TGX Precast Gels (Bio-Rad). Electrophoresis was run for 10-15 min at 80 V and then for 45 min at 120 V. The proteins were transferred to PVDF membranes using a Trans-Blot Turbo Transfer System (Bio-Rad). The membranes were blocked for 1 h with 5% milk in 0.05% Tween 20/TBS buffer and thereafter incubated with primary antibodies against GFAP (chicken, 1:30000, Abcam, ab4674) or vimentin (mouse, 1:1000, Dako, M0725) for 1 h at RT. A β-actin antibody (mouse, 1:2000, Santa Cruz, sc-47778) was used as a loading control. The membranes were incubated with the secondary antibodies goat anti-mouse IgG-HRP (goat, 1:2000, Santa Cruz, sc-2005) and goat anti-chicken IgY-HRP (goat, 1:30000, Invitrogen, A16054) for 1 h at RT. Then, the membranes were incubated with ECL Prime Western Blotting System reagent (Sigma-Aldrich) and imaged with a ChemiDoc XRS+ System (Bio-Rad). The band intensities were quantified with Image Lab (Bio-Rad) and compared to

that of the loading control. The full-length images of the membranes are presented in Supplementary Fig. S4.

MEA data analysis

Spike detection was performed by combining threshold-based spike detection and the stationary wavelet transform-based Teager energy operator (SWTTEO) algorithm [4, 5] embedded in custom MATLAB script. The data was filtered with an elliptic bandpass filter with a 200 Hz lower passband frequency and a 3000 Hz upper passband frequency. The threshold for spike detection was set to 4.5 × the estimate of the noise standard deviation. Burst analysis was performed utilizing the R package meaRtools [6], and for burst detection, the logISI algorithm was integrated into the analysis code [7] with a minor modification. The minimum number of spikes in a burst was set to five. Short bursts were merged when the ISIth was lower than 100 ms. A cutoff of 100 ms was applied as the minimum time required between bursts.

Fabrication of the microfluidics device

The device was produced from two PDMS parts: 1) a microfluidic device containing the cell compartments and microtunnels and 2) a medium reservoir part (Supplementary Fig. S7). The microfluidic device part was fabricated from PDMS (10:1 mass ratio with curing agent, SYLGARD 184, Dow Corning) using replica molding [8-10]. For the fabrication of the mold, multilayer SU-8 rapid prototyping methods were used: SU-8 5 (MicroChem) photoresist was used to produce the 3.5 µm high microtunnels, and SU-8 3050 photoresist was used to produce the 100 µm high cell compartments. The microfluidic device was separated from the replica using a ø 32 mm punch. To connect the cell compartments to the medium reservoirs and to enable cell plating, three inlets and one outlet (Supplementary Fig. S7) were punched in the device using a ø 3 mm punch. The medium

reservoir part was fabricated from an 8 mm thick PDMS sheet using a ø 32 mm punch. The medium reservoirs for each cell compartment were created with a ø 6 mm punch.

Fluidic isolation between the cell compartments

To demonstrate fluidic isolation between the cell compartments in the device, FITC-conjugated dextran particles (15-25 kDa) (TdB Consultancy, Uppsala, Sweden) were used. The particles (50 µM) were added to the astrocyte compartment, and their diffusion was evaluated for 1 h and 24 h at 37°C. The diffusion of the dextran particles into the axonal and neuronal compartments was visualized with an Olympus IX51 microscope equipped with an Olympus DP30BW camera (Olympus Corporation). To quantify the amounts of dextran particles in the neuronal soma and astrocyte compartments after 24 h, the absorbance of medium samples at 490 nm was measured using a NanoDrop 1000 (Thermo Fisher Scientific).

Assembly of the microfluidic platform and cell plating

The microfluidic devices and medium reservoirs were attached together and treated with oxygen plasma in a PICO plasma system (Diener Electronic, Germany) for 4 min at 30 W and 0.3 mbar pressure to make the cell compartments and microtunnels hydrophilic and to facilitate the supply of laminin in the microscale features. Thereafter, the devices were reversibly bonded on 250 µg/ml PO-coated coverslips (ø 30 mm), and the cell compartments and microtunnels were filled with 20 µg/ml LN521 via the inlets in the neuron and astrocyte compartments. Coating was performed overnight at 4°C. Neurons were seeded into the inlets of the neuronal compartments at a density of 150000 cells/cm^2, while 50000 astrocytes were seeded into the inlet of the astrocyte compartment. Cell plating on the astrocyte compartment is based on fluid flow, which enables astrocytes to settle in the cell area next to the microtunnels (Supplementary Fig. S7). The fluid flow is a result of differences in width

along the astrocyte compartment: the inlet side of the astrocyte compartment is wider than the outlet side of the astrocyte compartment.

References

1. Skottman, H. Derivation and characterization of three new human embryonic stem cell lines in Finland. *In Vitro Cell. Dev. Biol. Anim.* **46**, 206-209 (2010).

2. Hongisto, H., Ilmarinen, T., Vattulainen, M., Mikhailova, A. & Skottman, H. Xeno- and feeder-free differentiation of human pluripotent stem cells to two distinct ocular epithelial cell types using simple modifications of one method. *Stem cell research & therapy* **8**, 291 (2017).

3. Shi, Y., Kirwan, P., Smith, J., Robinson, H. P. & Livesey, F. J. Human cerebral cortex development from pluripotent stem cells to functional excitatory synapses. *Nat. Neurosci.* **15**, 86, S1 (2012).

4. Mayer, M. *et al*. Electrophysiological investigation of human embryonic stem cell derived neurospheres using a novel spike detection algorithm. *Biosens. Bioelectron.* **100**, 462-468 (2018).

5. Quiroga, R. Q., Nadasdy, Z. & Ben-Shaul, Y. Unsupervised spike detection and sorting with wavelets and superparamagnetic clustering. *Neural Comput* **16**, 1661-1687 (2004).

6. Gelfman, S. *et al*. meaRtools: An R package for the analysis of neuronal networks recorded on microelectrode arrays. *PLoS Comput. Biol.* **14**, e1006506 (2018).

7. Pasquale, V., Martinoia, S. & Chiappalone, M. A self-adapting approach for the detection of bursts and network bursts in neuronal cultures. *J. Comput. Neurosci.* **29**, 213-229 (2010).

8. Duffy, D. C., McDonald, J. C., Schueller, O. J. & Whitesides, G. M. Rapid Prototyping of Microfluidic Systems in Poly(dimethylsiloxane). *Anal. Chem.* **70**, 4974-4984 (1998).

9. Taylor, A. M. *et al*. Microfluidic Multicompartment Device for Neuroscience Research. *Langmuir* **19**, 1551-1556 (2003).

10. Ristola, M. *et al*. A compartmentalized neuron-oligodendrocyte co-culture device for myelin research: design, fabrication and functionality testing. *J Micromech Microengineering* **29**, 065009 (2019).
